# Taurine as a water structure breaker and protein stabilizer

**DOI:** 10.1007/s00726-017-2499-x

**Published:** 2017-10-17

**Authors:** P. Bruździak, A. Panuszko, E. Kaczkowska, B. Piotrowski, A. Daghir, S. Demkowicz, J. Stangret

**Affiliations:** 1 Department of Physical Chemistry, Chemical Faculty, Gdańsk University of Technology, Narutowicza 11/12, 80-233 Gdańsk, Poland; 2 Department of Organic Chemistry, Chemical Faculty, Gdańsk University of Technology, Narutowicza 11/12, 80-233 Gdańsk, Poland

**Keywords:** Taurine, Hydration, Protein interactions, DSC calorimetry, FTIR spectroscopy, DFT calculations

## Abstract

The enhancing effect on the water structure has been confirmed for most of the osmolytes exhibiting both stabilizing and destabilizing properties in regard to proteins. The presented work concerns osmolytes, which should be classified as “structure breaking” solutes: taurine and *N,N,N*-trimethyltaurine (TMT). Here, we combine FTIR spectroscopy, DSC calorimetry and DFT calculations to gain an insight into the interactions between osmolytes and two proteins: lysozyme and ubiquitin. Despite high structural similarity, both osmolytes exert different influence on protein stability: taurine is a stabilizer, TMT is a denaturant. We show also that taurine amino group interacts directly with the side chains of proteins, whereas TMT does not interact with proteins at all. Although two solutes weaken on average the structure of the surrounding water, their hydration spheres are different. Taurine is surrounded by two populations of water molecules: bonded with weak H-bonds around sulfonate group, and strongly bonded around amino group. The strong hydrogen-bonded network of water molecules around the amino group of taurine further improves properties of enhanced protein hydration sphere and stabilizes the native protein form. Direct interactions of this group with surface side chains provide a proper orientation of taurine and prevents the ${\text{SO}}_{3}^{ - }$ group from negative influence. The weakened ${\text{SO}}_{3}^{ - }$ hydration sphere of TMT breaks up the hydrogen-bonded network of water around the protein and destabilizes it. However, TMT at low concentration stabilize both proteins to a small extent. This effect can be attributed to an actual osmophobic effect which is overcome if the concentration increases.

**Electronic supplementary material:**

The online version of this article (doi:10.1007/s00726-017-2499-x) contains supplementary material, which is available to authorized users.

## Introduction

Many organisms living in harsh environmental conditions developed different mechanisms to overcome the effects of adverse surrounding. One of them is an accumulation of small organic compounds commonly called osmolytes (Yancey 2001, 2004; Yancey et al. 2002; Auton et al. 2011; Singh et al. 2011; Panuszko et al. 2009, 2016; Bruździak et al. 2012, 2016) which, besides the osmotic pressure regulatory function, have an impact on macromolecules’ stability. Considering the chemical structure, this group of molecules includes: polyols, amino acids, amines, urea and its derivatives, etc. This article is devoted to taurine and its influence on water structure and protein stability.

Taurine is a β-amino acid, however, instead of the carboxylic group it has the sulfonate one. This functional group is more acidic than the carboxylate one and this property [*pK*_*a*_ < 0–1.5, various values are available in literature (Madura et al. 1997)] makes taurine almost completely zwitterionic (${\text{NH}}_{3}^{ + }$–CH_2_–CH_2_–${\text{SO}}_{3}^{ - }$) over the physiological pH range. Taurine can be found in many animal tissues, particularly in brain (Okumura et al. 1960; Wade et al. 1988), liver, muscle (Lombardini 1996; Schaffer et al. 2010), and kidney (Chesney et al. 2010; Jackson-Atogi et al. 2013; Suliman 2002; Michalk et al. 2003; Khan et al. 2013); however, it is not incorporated into protein structures. Major functions of taurine include osmoregulation and tissue protection (Shiny et al. 2005; Uchida et al. 1991; Ando et al. 2012; Schaffer et al. 2003), bile salts synthesis (Murakami 2015; Jacobsen and Smith 1968; Huxtable 1992), modulation of channel Ca^2+^ activity, neuroinhibition, and energy storage (Jacobsen and Smith 1968). Its ability to regulate osmotic pressure originates from its inertness at physiological pH, lack of metabolic function in most cells and poor diffusion through the cellular membrane. Unlike other osmolytes, taurine’s influence on proteins is poorly described in literature, although it is extensively used as a popular food and beverage additive (Chesney 1985; Chesney et al. 1998; Hansen 2001; Mora-Rodriguez and Pallarés 2014). Few papers deal with the subject of taurine–macromolecule interactions and confirm taurine’s stabilizing influence on the native structure of proteins (Abe et al. 2015; Arakawa and Timasheff 1985; Pieraccini et al. 2007). Despite significant advances in understanding of osmolytes’ effects on protein stability, the mechanism of this stabilization is not clear. There are several hypothetical models describing phenomenon of such a stabilization. The most popular one concerns the osmolyte exclusion mechanism (Auton et al. 2011), but it does not seem to be universal. On the other hand, the theory concerning indirect impact of osmolyte on macromolecule through affected water molecules seems to be more general (Panuszko et al. 2016). Thus, to understand the effect of osmolytes on protein stability it is essential to understand their influence on the water structure and interactions between water molecules.

In this work, we want to present a comprehensive study on the influence of taurine protein thermal stability and its interactions in protein solutions. Taurine’s derivative—*N,N,N*-trimethyltaurine (TMT)—turned out to be necessary to fully explain some taurine-induced phenomena, thus all experiments and analysis were performed for that compound as well. Density functional theory calculations of various systems containing taurine and TMT support experimental findings and help to explain them. FTIR vibrational spectroscopy was used also to study taurine’s effect on the structure of water as a function of temperature. This technique is an ideal tool to observe subtle changes taking place in the net of hydrogen-bonded water. The stretching band *ν*_*OD*_ of the HDO molecule, isotopically diluted in H_2_O, was utilized as a molecular probe of interactions in taurine and TMT solutions. HDO spectra are mostly free of experimental and interpretative problems connected with H_2_O spectra. The structural and energetic state of water molecules around these compounds was investigated on the basis of the band shape of affected HDO spectra. The obtained spectral results were compared with DFT calculated structures of small hydrated complexes using the polarizable continuum model of solvation.

## Experimental

### Chemicals and solutions

Taurine (99%, Alfa Aesar, Haverhill, MA), ubiquitin from bovine erythrocytes (Sigma-Aldrich, Saint Louis, MO) and D_2_O (isotopic purity 99.9%, Aldrich) were used as supplied. *N,N,N*-trimethyltaurine (TMT) was prepared from 2-bromoethyltrimethylammonium bromide and sodium sulfite as described by Barnhurst (1961). Hen egg white lysozyme (Sigma-Aldrich, Saint Louis, MO) was used with further purification which included dialysis against deionized water and lyophilization, see Panuszko et al. (2012) for details. No additional buffers or salts were added to any solutions to get a clear picture of interactions in solutions of taurine, TMT, water and proteins.

For ATR-FTIR studies six series of taurine–lysozyme and five of taurine–ubiquitin, TMT–lysozyme, and TMT–ubiquitin systems were prepared with different but fixed concentrations of taurine (0.0, 0.1, 0.2, 0.3, 0.4 mol∙dm^−3^ of taurine or TMT and additional 0.5 mol∙dm^−3^ in the case of taurine–lysozyme system). In each series, six spectra of taurine–protein systems were collected in which the concentration of protein varied in the range of 0.0–13.0 or 0.0–16.0 mmol dm^−3^ of lysozyme or ubiquitin, respectively.

In the case of DSC studies five or six solutions of lysozyme or ubiquitin, respectively, were prepared with constant protein concentration of 1.5 mg mL^−1^ and various taurine or TMT concentrations in the range of 0.0–0.4 mol dm^−3^.

All concentrations of osmolyte, protein and water, for the above-mentioned experiments, were calculated on the basis of appropriate weights of all solutions constituents, densities of osmolyte solutions, and partial specific volumes of proteins [0.703 and 0.675 cm^3^ g^−1^ for lysozyme and ubiquitin, respectively (Imai et al. 2007; Lee and Timasheff 1974)]. The solution preparation procedure for FTIR measurements of HDO spectra has been described in section S1 (ESM).

### Methods of interaction studies in protein–osmolyte–water systems

#### Differential scanning calorimetry

Calorimetric experiments were performed by means of the 6300 nano-DSC III calorimeter (TA Instruments, New Castle, DE) equipped with a capillary platinum cell (V 0.299 mL) with a scanning rate of 1 K min^−1^. The temperature range depended on the protein native thermal stability in such experimental conditions (25–95 or 35–110 °C in the case of lysozyme or ubiquitin, respectively). The denaturation temperature of a protein was understood as the maximum of baseline-corrected DSC curve. In the case of lysozyme, the baseline fitting was straightforward (linear and quadratic polynomials in pre- and post-*T*_m_ the regions of thermograms, respectively) and the error of denaturation temperature was estimated as ± 0.1 °C. Ubiquitin denaturation in such experimental conditions was not typical and both pre- and post-*T*_m_ regions were fitted with quadratic curves. The nonstandard shape of transition curves in that case increased the uncertainty of *T*_m_ value, here estimated as ± 0.5 °C. Data was collected with DSCRun software (TA Instruments, New Castle, DE) and analyzed with NanoAnalyze software (TA Instruments, New Castle, DE). Baseline-corrected thermograms and their first derivatives are presented in ESM in Figs. S7–S27.

#### ATR-FTIR spectroscopy

All spectra were recorded using the Nicolet 8700 FTIR spectrometer (Thermo Scientific, Waltham, MA) equipped with a single-reflection diamond crystal Golden Gate ATR accessory (Specac Ltd., Orpington, Great Britain). The temperature during measurements was kept at 25 ± 0.1 °C using an electronic temperature controller (Specac Ltd., Orpington, Great Britain). For each spectrum 512 scans were collected with resolution of 2 cm^−1^. The spectrometer and ATR accessory were purged with dry nitrogen to diminish the water vapor contamination. All FTIR spectra were handled and analyzed using the commercial software: OMNIC (Thermo Scientific, Waltham, MA), GRAMS/32 (Galactic Industries Corporation, Salem, NH), and Matlab (the MathWorks, Natic, MA) with Factor Analysis Toolbox (Applied Chemometrics Inc., Sharon, MA).

A variant of the difference spectra method supported by a chemometric method of spectral data analysis was employed to isolate spectra of taurine and TMT affected by the presence of proteins (Bruździak et al. 2010; Schostack and Malinowski 1989; Malinowski 1982). Also, after determination of the number of taurine or TMT molecules affected by one protein molecule the preferential interaction coefficients were calculated according to the method presented in Bruździak et al. (2015). Both these methods are briefly described in sections S2 and S3 of ESM.

The chemometric method of “affected spectrum” determination on a single spectral series usually gives several possible *N* numbers and corresponding “affected spectra”. The lowest *N* number (*N*_1_) can be interpreted as the number of the most “affected” molecules. Higher *N* values (*N*_2_ *, N*_3_ *… N*_i_) correspond to “affected spectra” of those molecules which are also less affected. It should be kept in mind that the *N*_1_ subset of molecules belongs to *N*_2_ *, N*_2_ is a member of *N*_3_ set, and so on. These subsets can be identified and characterized with the number of “affected molecules” and appropriate spectra. Unfortunately, due to experimental and mathematical uncertainty of a single analysis on such a spectral series may lead to false negative or false positive indications of *N* number. In this paper, we use an upgraded method of chemometric analysis which allows to extract information from *N*_1_ *, N*_2_ *… N*_i_ subsets and to unify *N* numbers (i.e., to reduce error of *N* determination) and, in consequence, affected spectra if a few series of spectroscopic data are available. The method is described in detail in section S4 of ESM.

#### DFT calculations: interactions of taurine and TMT with model molecules

All DFT calculations concerning taurine or TMT interactions with molecular models of proteins were performed according to the following scheme: (1) pre-optimization in gas phase of predicted structures and complexes with HF method and aug-cc-pVDZ basis set; (2) proper optimization of gas-phase structures with M06-2X (Zhao and Truhlar 2008) density functional and aug-cc-pVTZ basis set; (Kendall et al. 1992) (3) optimization and frequency calculation of resultant structures within the conductor-like polarizable continuum model (CPCM) (Barone and Cossi 1998; Cossi et al. 2003) of the solvent (water) with cavities built with UFF radii. All calculated structures exhibited no negative vibration frequencies; thus, all of them were taken as structures corresponding to local energetic minima. In the case of complex structures, the basis set superposition error (BSSE) was estimated on the basis of CPCM-optimized structures by the counterpoise method (Simon et al. 1996; Boys and Bernardi 1970). All calculations were performed with the Gaussian 09v.D1 (Gaussian Inc., Wallingford, CT) (Frisch et al. 2009) software available at the Academic Computer Center in Gdansk (TASK), analyzed and visualized with Avogadro software version 1.1.1. (Hanwell et al. 2012).

### Methods of interactions studies in osmolyte–water systems

#### FTIR spectroscopy

FTIR spectra of aqueous solutions of taurine and TMT (resolution of 4 cm^−1^, 500–5000 cm^−1^, 128 independent scans) were recorded on Nicolet 8700 spectrometer (Thermo Electron Co.) at different temperatures (from 25 to 75 °C with 10 °C step). A thermocouple was inserted into the cell to monitor its temperature. The temperature was maintained with accuracy of ± 0.1 °C. Dry nitrogen was used to purge spectrometer’s interior. A liquid transmission cell (model A145, Bruker Optics) was used with two CaF_2_ windows separated by Teflon spacers with path length of 0.03 mm (determined interferometrically).

#### Analysis of HDO spectral data

The difference spectra method was applied to extract the solute-affected HDO spectrum on the basis of spectra series measured for different molalities of aqueous solutions (Stangret 1988; Stangret and Gampe 1999). An assumption was made that the water in solution can be divided into two additive contributions: the “bulk” water (identical to pure water) and “affected” water, the qualities of which have been affected by interactions with the solute. The method was described in detail in Stangret (1988), Stangret and Gampe (1999) and Śmiechowski and Stangret (2010), and some of the most basic information are included in section S1 (ESM).

All spectra have been handled and analyzed using the commercial computer software: OMNIC (Thermo Electron Corporation), GRAMS/32 (Galactic Industries Corporation, Salem, NH), and RAZOR (Spectrum Square Associates, Inc., Ithaca, NY) run under GRAMS/32.

#### DFT calculations of taurine and TMT hydration complexes

The optimized geometries of small hydrated complexes of taurine and TMT were calculated using the density functional theory (DFT). The B3LYP functional (Becke 1993; Lee et al. 1988) with 6-311++G(d,p) basis set was used (Krishnan et al. 1980; Frish et al. 1984). The polarizable continuum solvation model (PCM) of self-consistent reaction field theory (SCRF) was used to simulate a bulk water environment (Cossi et al. 2002; Mennucci and Tomasi 1997). The structures of the hydrated complexes were initially optimized in the gas phase and next re-optimized within the PCM model. All calculations have been carried out using the GAUSSIAN 09v.D1 program package (Frisch et al. 2009). The program HyperChem 8 (Hypercube, Gainesville, FL) was applied to the preparation of input data and for visualization of computed results.

## Results and discussion

### DSC results

**Fig. 1:**
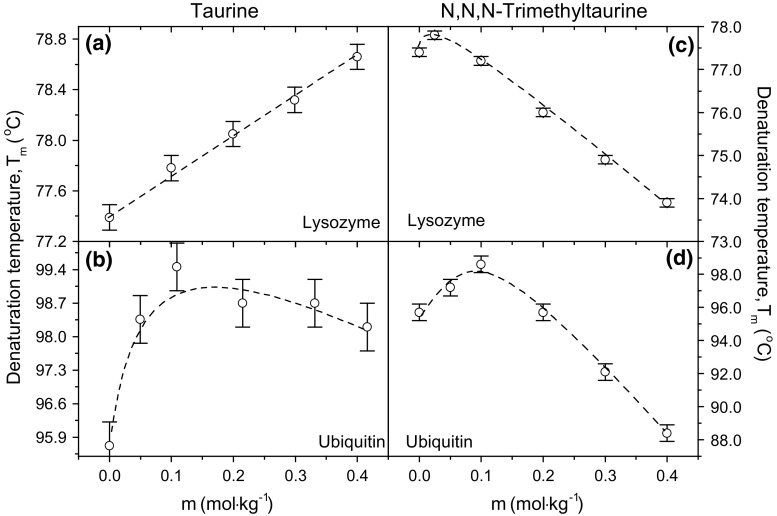
The dependencies of lysozyme and ubiquitin denaturation temperature versus taurine (**a**, **b**) or TMT (**c**, **d**) molality. Dashed lines are presented only for visual purposes

In cases of both lysozyme and ubiquitin, taurine acts as a stabilizer increasing the protein denaturation temperature (Fig. 1a, b, respectively). However, these proteins do not react the same way to the presence of osmolyte. Lysozyme gradually increases its denaturation temperature with osmolyte’s molality, while ubiquitin is stabilized the most with a small amount of taurine. Further increase of its molality causes a rather small decrease in ubiquitin’s thermal stability. This does not change the fact that in the whole accessible concentration range of taurine both proteins are stabilized.

*dT*_m_ */dm* parameter for lysozyme in the presence of taurine, i.e., the slope of *T*_m_ vs. *m* graph, is equal to 3.1 K mol^−1^. Such a value characterizes taurine as a rather moderate stabilizer in the case of lysozyme. In the case of ubiquitin calorimetric data, this parameter cannot be derived directly from an appropriate T_m_ vs*. m* dependence due to nonlinearity of melting temperature data. However, the first additive of taurine to a ubiquitin solution (0.05 mol kg^−1^) results in a melting temperature rise of 2.7 K, which gives (*dT*_m_ */dm*)_m→0_ ≈ 54 K mol^−1^. Such a high value of ubiquitin’s melting temperature increase in the presence of taurine indicates that stabilizing properties of an osmolyte are highly dependent on the protein type.

Same parameters for lysozyme and ubiquitin denaturation in the presence of TMT indicate that this compound is a good denaturing agent (Fig. 1c, d, respectively). The slopes of the *T*_m_ vs. *m* graphs for higher TMT concentrations lead to a conclusion that the presence of three methyl groups causes the melting temperature to drop by ca. 12 or 40 °C at each mol of TMT per kg of water for lysozyme and ubiquitin, respectively. However, at low molalities both proteins are stabilized to a small extent which can be caused by the osmophobic effect, i.e., TMT is excluded from the protein surrounding if its amount is small. This effect is overcome at higher TMT concentrations when other interactions in solution take place. The nature of these interactions remains indirect, as ATR-FTIR results indicate, and is connected with properties of water surrounding TMT and proteins.

### ATR-FTIR results

#### Changes in the band shape: N–H bending vibrations

**Fig. 2:**
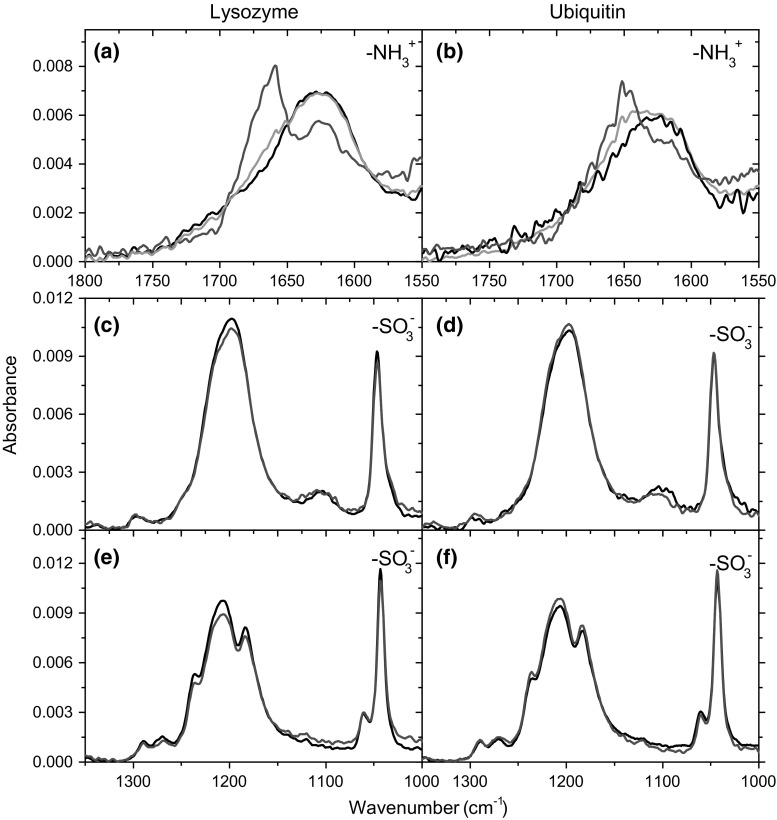
**a**, **b** Bulk (black), highly (red) and less (green) protein-affected spectra of taurine in the spectral region of NH bending vibration. **c**, **d** Bulk (black) and protein-affected (red) FTIR spectra of taurine in the spectral region of ${\text{SO}}_{3}^{ - }$ stretching vibrations. **e**, **f** Bulk (black) and protein-affected (red) FTIR spectra of TMT in the spectral region of ${\text{SO}}_{3}^{ - }$ stretching vibrations. Area of all spectra were normalized to a unitary area

The lysozyme- and ubiquitin-affected spectra of taurine in the region of N–H bending vibrations are significantly different from the bulk spectrum of taurine in pure aqueous solution (Fig. 2a, b, respectively). However, they are similar to each other. The peak at ca. 1625 cm^−1^, which can be attributed to bending vibrations of ${\text{NH}}_{3}^{ + }$, is accompanied with an additional one with the maximum at higher (1660–1650 cm^−1^) wavenumbers. The presence of the additional peak with distinct spectral properties suggests that an interaction occurs involving hydrogen atom of the ${\text{NH}}_{3}^{ + }$ group.

**Fig. 3:**
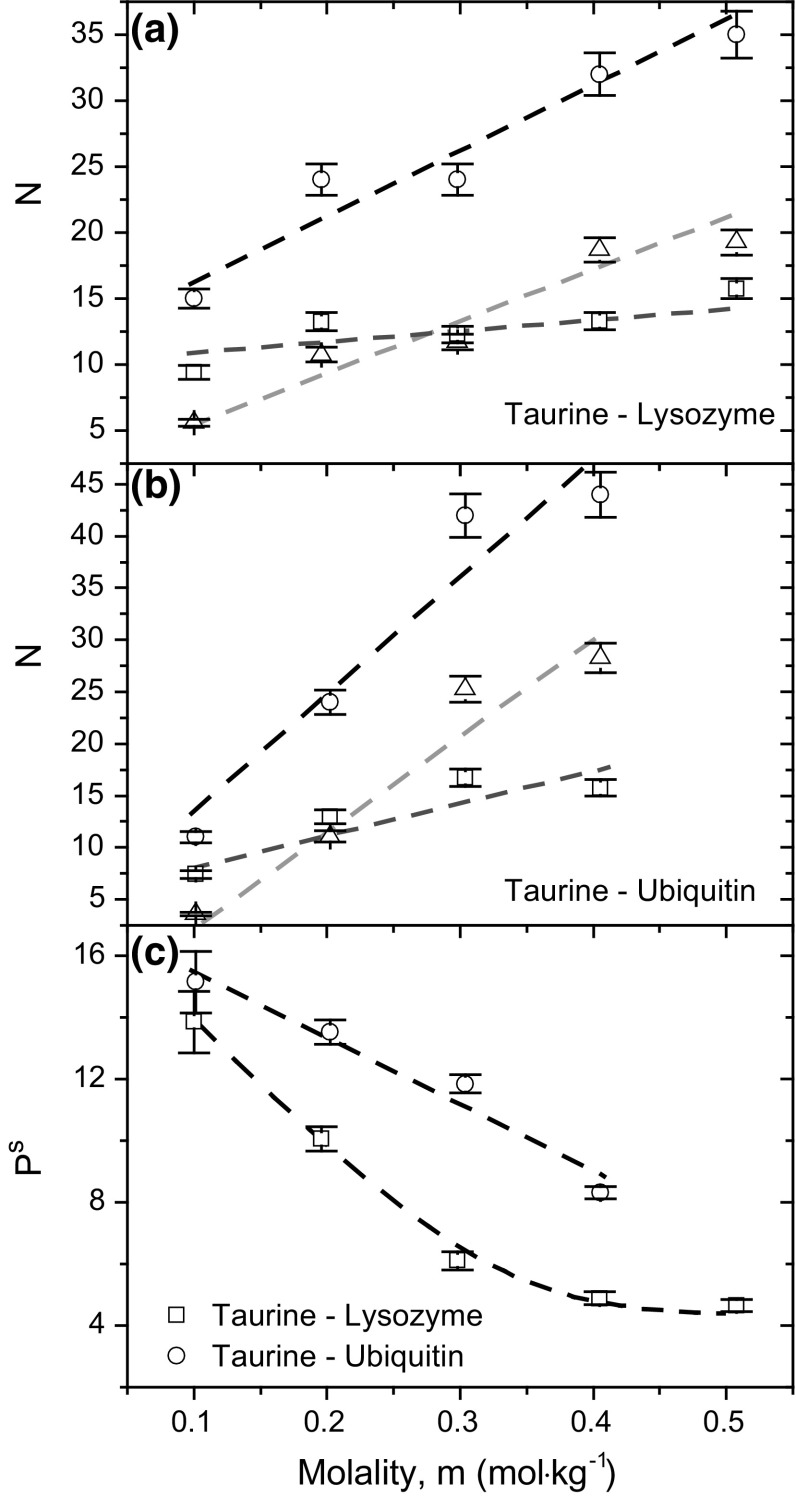
**a**, **b** Affected numbers, *N*, of taurine molecules at various molalities (circles) in solutions of **a** lysozyme and **b** ubiquitin decomposed into two differently affected contributions: highly affected one (squares) and less affected one (triangles). **c** Preferential interaction coefficients, derived from spectroscopic data, corresponding to highly affected taurine molecules in these systems. Dashed lines are presented only for visual purposes and have no physical meaning

Less affected taurine spectra, calculated with the aid of chemometric method presented in ESM, exhibit high similarity to the bulk taurine spectrum with only a small shoulder in the place of maximum peak of highly affected taurine molecules. Thus, such interactions between taurine and both proteins are weak. These results also indicate that the number of taurine molecules highly affected by proteins changes to a smaller extent than the number of less affected molecules (Fig. 3a, b). Thus, a limited number of interaction centers on the protein surface may exist where taurine molecules creates direct and strong bonds, as the shape of highly affected spectra suggests. Similar conclusion can be drawn from the analysis of spectral preferential interaction coefficients (Fig. 3c). It reaches the highest value in the case of first low-concentration solution of taurine, where the first average affected number is close to the first highly affected number of taurine molecules. The coefficient decreases in higher taurine concentrations, but it does not reach 1 in the whole possible range of concentrations which indicates that is not excluded in the whole possible taurine concentrations in such a system.

#### Changes in the band shape: S–O stretching vibrations

In contrast to changes in the shape of N–H bending vibration bands, ${\text{SO}}_{3}^{ - }$ band shape of lysozyme- and ubiquitin-affected spectra of taurine does not change significantly (Fig. 2c, d, respectively). These spectra of taurine are almost identical to the bulk spectrum of taurine in solution. In comparison to the ${\text{NH}}_{3}^{ + }$ group, no direct interactions between this chemical group and the protein surface are present in a solution. Such results indicate that taurine molecule is oriented with the ${\text{NH}}_{3}^{ + }$ group towards the protein surface and the ${\text{SO}}_{3}^{ - }$ group is facing the bulk solution.

In the case of lysozyme- and ubiquitin-affected TMT, the band shape of affected spectra does not change significantly, and as in the case of taurine, only changes in intensities are visible (Fig. 2e, f). Similarly, no strong direct interactions in solutions are made between the ${\text{SO}}_{3}^{ - }$ group of TMT and the protein surface.

#### DFT calculations: interactions of taurine or TMT with model molecules

**Fig. 4:**
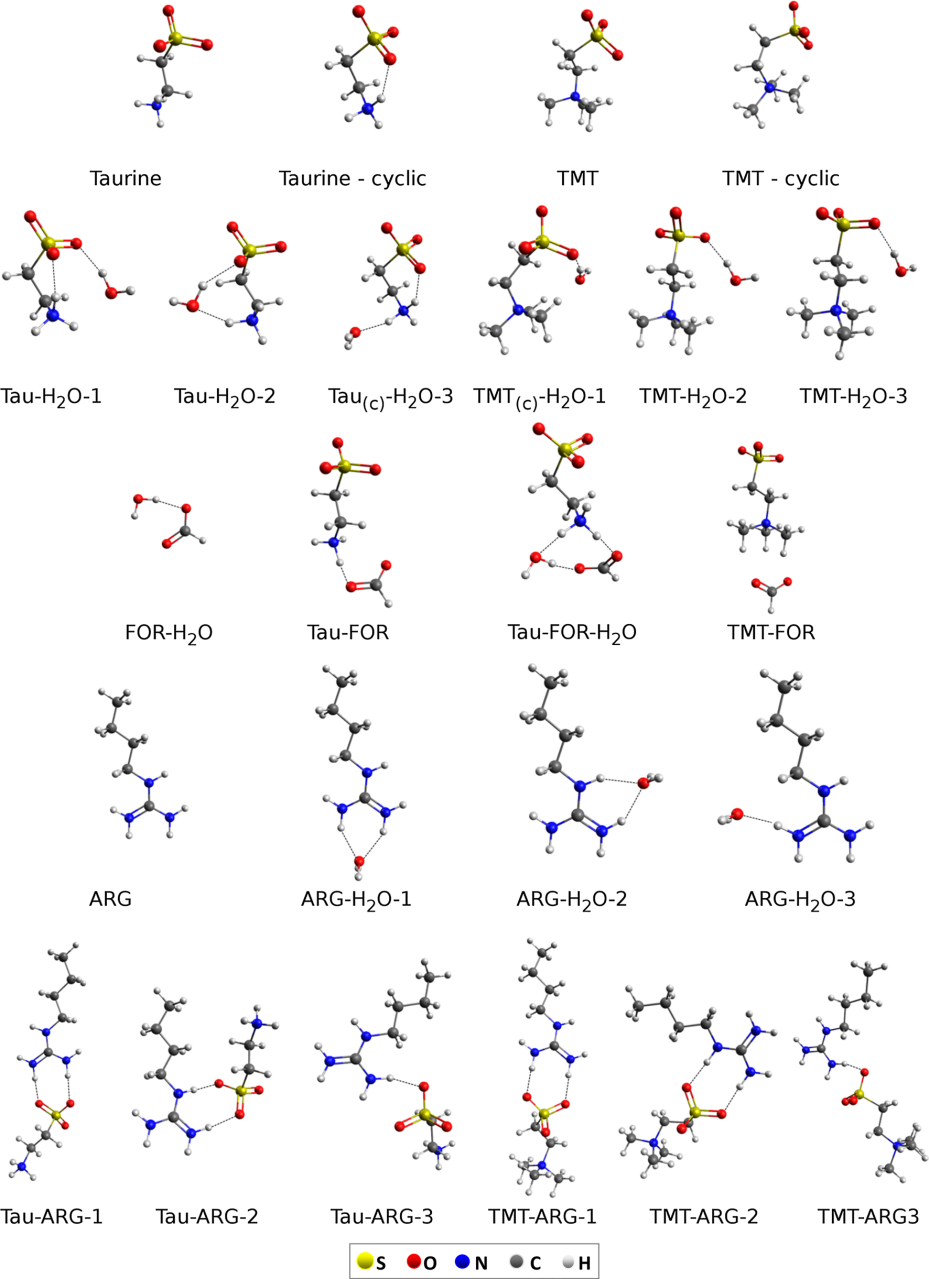
Optimized structures of taurine, TMT, ARG, FOR and H_2_O for which DFT energies and frequencies were calculated (M06-2X/aug-cc-pVTZ with CPCM water solvent model). Names of all molecules and complexes correspond to the ones presented in Table 1. **c** Represents cyclic form of taurine

To gain an insight into the specific intermolecular interactions in aqueous solutions of taurine or TMT with proteins, we performed a series of DFT calculations. Various complexes of taurine or TMT, water and model molecules were studied within the CPCM model (all optimized structures are presented in Fig. 4). Model molecules included: formate anion (FOR), 1-*n*-butyl-guanidine (ARG), serving as models of acidic surface amino acid residues, and surface arginine residue, respectively. We tried to use butyric acid as a model of acidic amino acid residue, however, in such a situation one of amino protons of taurine always shifted to the carboxylic group of the acid, regardless of DFT methods, basis sets or solvent models used in calculations. That way, no stable zwitterionic form of taurine was formed, which is unlikely in aqueous solutions due to acid dissociation constant values of both ${\text{NH}}_{3}^{ + }$ and SO_3_H chemical groups of taurine, pKa 9.08–9.06 and 1.5, respectively (Madura et al. 1997; Huxtable 1987). Energies of all complexes, including the BSSE error, are presented in Table 1. Under the term “excessive hydrogen bonds” in complex structures we mean any hydrogen bond that is not present in any of the monomer form, i.e., such hydrogen bond which result from monomers’ interactions.

**Table 1:** Results of DFT (M06-2X/aug-cc-pVTZ, CPCM) energies calculations of various complexes of taurine, TMT, water, ARG, and FOR

	E(el. + ZPC)^a^ hartree	BSSE^b^ (kJ mol^−1^)	∆*E* ^c^ (kJ mol^−1^)	H-bonds^d^	*E*_H-bond_^e^ (kJ mol^−1^)
Water (H_2_O)	−76.415491	–	–	–	–
Taurine—linear form (Tau)	−758.935694	–	–	–	–
Taurine—cyclic form (Tau_(c)_)	−758.944938	–	–	–	–
Tau_(c)_-H_2_O-1	−835.369499	1.10	−23.09	1	−23.09
Tau-H_2_O-2	−835.368468	1.00	−44.38	2	−22.19
Tau_(c)_-H_2_O-3	−835.368215	0.57	−19.87	1	−19.87
TMT—linear form	−876.754560	–	–	–	–
TMT—cyclic form (TMT_(c)_)	−876.755305	–	–	–	–
TMT_(c)_-H_2_O-1	−953.177806	1.06	−17.34	1	−17.34
TMT-H_2_O-2	−953.180077	0.93	−25.39	1	−25.39
TMT-H_2_O-3	−953.179990	0.94	−25.15	1	−25.15
Formate ion (FOR)	−189.296181	–	–	–	–
FOR-H_2_O	−265.721846	0.46	−26.25	1	−26.25
1-*n*-Butyl-guanidine (ARG)	−362.874316	–	–	–	–
ARG-H_2_O-1	−439.298845	0.67	−23.06	2	−11.53
ARG-H_2_O-2	−439.298730	0.79	−22.64	2	−11.32
ARG-H_2_O-3	−439.296212	0.72	−16.09	1	−16.09
Tau-ARG-1	−1121.827313	1.64	−43.79	2	−21.90
Tau-ARG-2	−1121.827917	2.28	−44.73	2	−22.37
Tau-ARG-3	−1121.822325	1.65	−30.68	1	−30.68
Tau-FOR	−948.260957	1.01	−75.34	1	−75.34
Tau-FOR-H_2_O	−1024.691705	1.76	−92.14	3	−30.71
TMT-ARG-1	−1239.646238	1.71	−43.87	2	−21.94
TMT-ARG-2	−1239.646906	2.06	−45.28	2	−22.64
TMT-ARG-3	−1239.642608	1.69	−34.36	2	−17.18
TMT-FOR	−1066.061522	0.84	−27.47	–	–

All descriptions of complexes are in agreement with structures presented in Fig. 4

^a^Sum of electronic and zero point energies, ^b^Basis set superposition error, ^c^Energy of interaction, BSSE included, ^d^Number of excessive hydrogen bonds in a complex, ^e^Energy of interaction per one excessive hydrogen bond

Calculated energies per one excessive hydrogen bond support experimental results. Values of energies of taurine complexes with various model compounds indicate that interactions of the osmolyte are favorable only in the case of ${\text{NH}}_{3}^{ + }$ group. Highest interaction energy values are observed in the case of taurine interacting directly with formate anion (with and without water molecule in a complex). Energies of interactions between sulfonate group and model compounds are comparable with energies of taurine–water interactions.

Analogous results for TMT indicate that this taurine derivative does not favor any interactions with molecules other than water. The energies of TMT–water interactions predicted with M06-2X method are even stronger than in the case of taurine. All of these findings show that TMT in solution prefers to avoid protein surface and rather tends to keep its hydration shell intact.

Vibration frequencies calculated with M06-2X method are very accurate and correspond to the experimental ones (Table 2). Vibrations of sulfonate and ammonium groups’ bonds calculated with the method allow to predict changes that should be observed in FTIR spectrum of real complexes. Such a change is present in both protein-affected taurine spectrum in the region of amide bands and in calculated spectrum of taurine in complex with FOR. Shifts in band positions should be visible in the protein-affected taurine and TMT spectra in the range of sulfonate group vibration bands, as predicted by DFT calculations, but none of them can be observed. A conclusion can be made that in aqueous solution taurine and TMT does not interact directly with protein through the sulfonate group.

**Table 2:** Results of DFT (M06-2X/aug-cc-pVTZ, CPCM) frequency calculations of various complexes of taurine, TMT, ARG, and FOR

Taurine^a^	Taurine	Taurine-FOR	Taurine-ARG^b^
1047 ($\nu_{s}$ ${\text{SO}}_{3}^{ - }$)	1052	1050	1052
1196 ($\nu_{as}$ ${\text{SO}}_{3}^{ - }$)	1214	1208	1194
1219 ($\nu_{as}$ ${\text{SO}}_{3}^{ - }$)	1222	1214	1234
1518 (*δ* ${\text{NH}}_{3}^{ + }$)	1526	1584	1531
1625 (*δ* ${\text{NH}}_{3}^{ + }$)	1654	1643	1658
1655 (*δ* ${\text{NH}}_{3}^{ + }$)		1670	

TMT^a^	TMT	TMT-FOR	TMT-ARG^b^
1043 ($\nu_{s}$ ${\text{SO}}_{3}^{ - }$)	1050	–	1051
1183 ($\nu_{as}$ ${\text{SO}}_{3}^{ - }$)	1190	–	1182
1207 ($\nu_{as}$ ${\text{SO}}_{3}^{ - }$)	1218	–	1206
1237 ($\nu_{as}$ SO_3_^-^)	1228	–	1242

Only structures with intermolecular hydrogen bonds taken into account. ^a^Average experimental values corresponding to taurine and TMT affected by proteins; the spectrum of bulk taurine does not exhibit 1655 cm^−1^ band. ^b^Given wavenumbers are average values over all appropriate complex structures. Wavenumbers in bold highlight the most important changes corresponding to taurine interactions

### FTIR results

#### Interpretation of affected water spectra

**Fig. 5:**
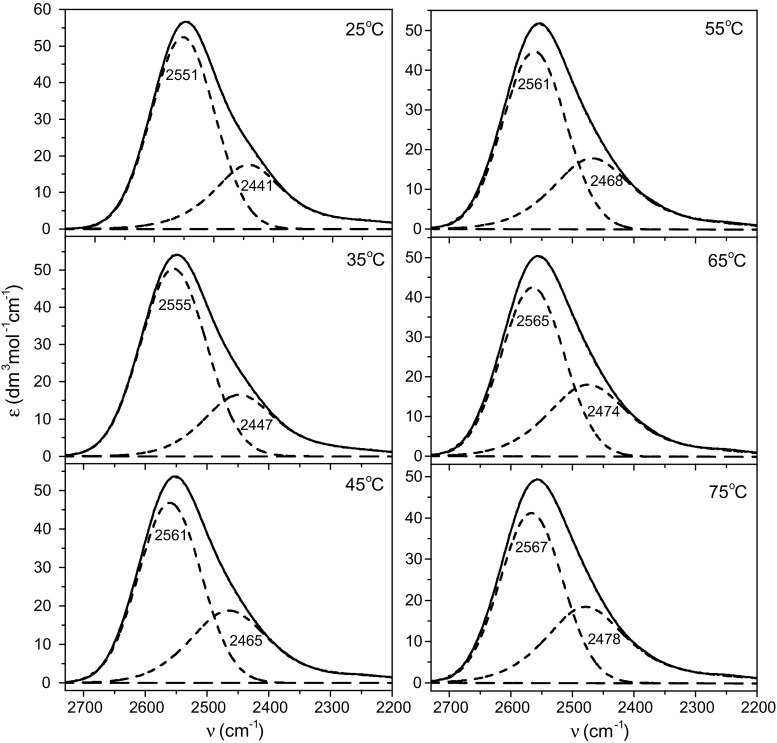
TMT-affected spectra for all studied temperatures decomposed into component bands. Solid line: original affected spectrum; dotted line: sum of the component bands (covered by the solid line of the original spectrum); dashed line: OD component band

**Fig. 6:**
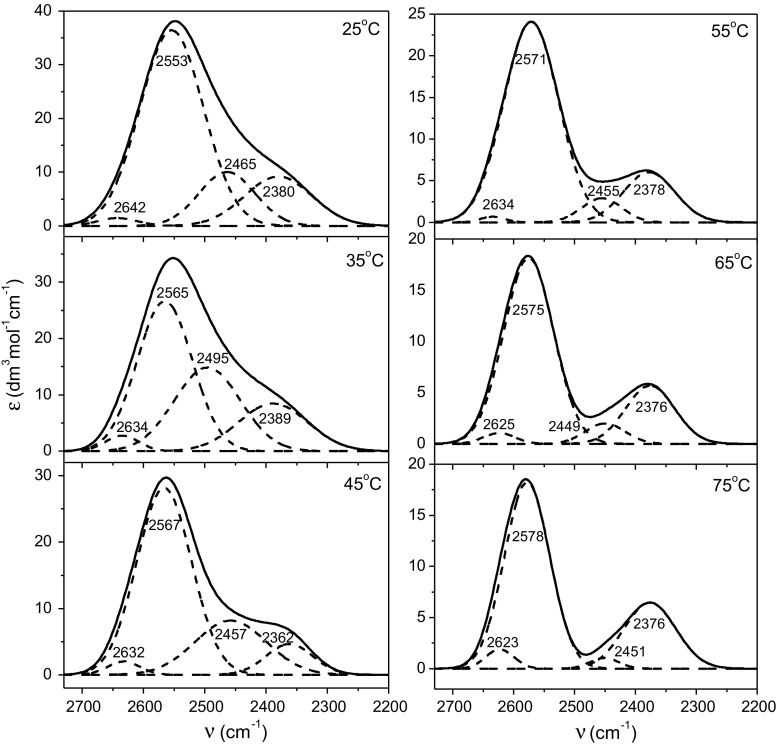
Taurine-affected spectra for all studied temperatures decomposed into component bands. Solid line: original affected spectrum; dotted line: sum of the component bands (covered by the solid line of the original spectrum); dashed line: OD component band

**Fig. 7:**
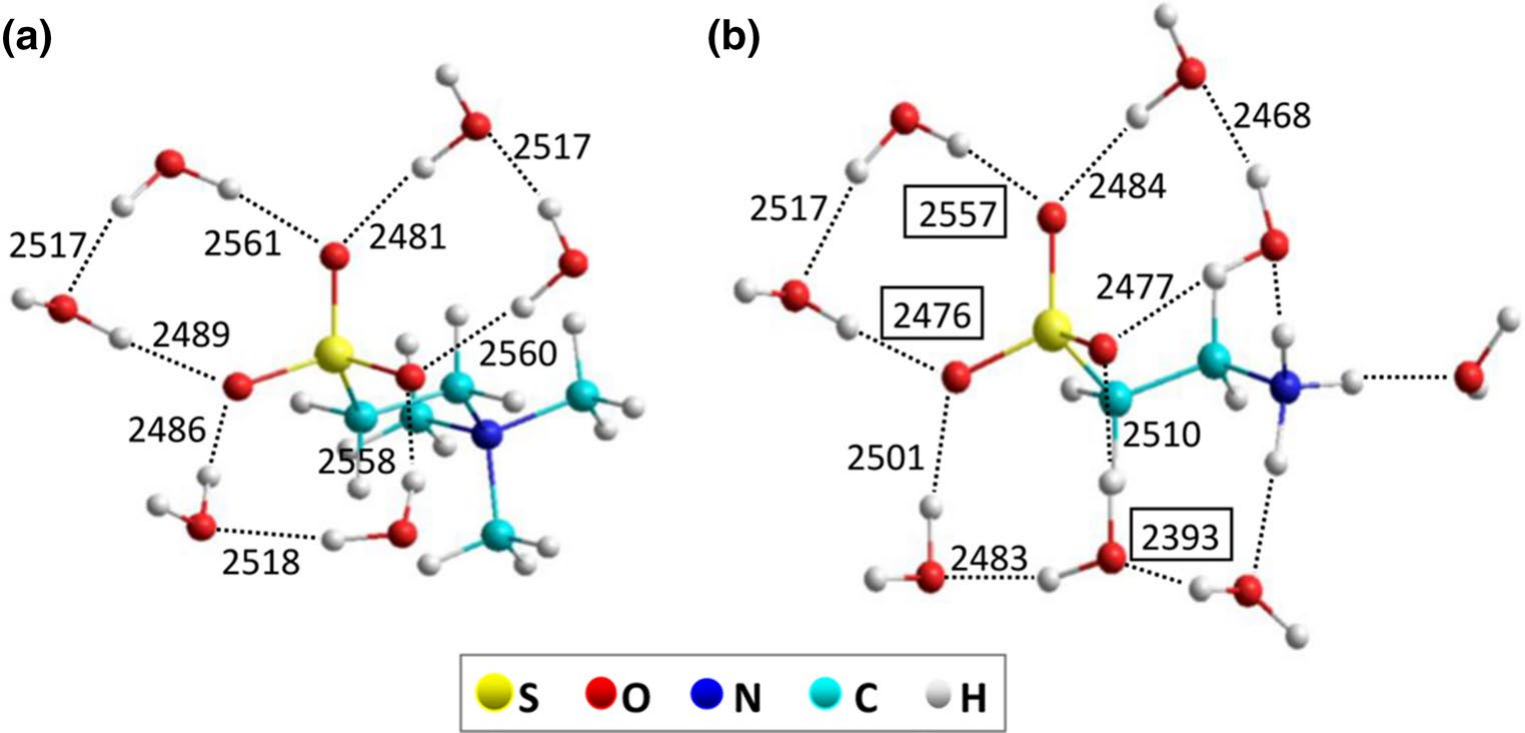
The optimized structures of hydrated complexes of **a** TMT and **b** taurine calculated in the PCM model and corresponding vibrational frequencies (cm^−1^) obtained from transformation of interatomic oxygen–oxygen distances (*R*_OO_) to the OD band position of HDO (*ν*_OD_) with the aid of the empirical relations (Eq. 1). Positions of the OD bands visible in the taurine-affected water spectra (Fig. 6) are put in frames. Hydrogen bonds indicated by dashed lines

Details concerning the analysis and interpretation of the solute-affected water spectra have been described in section S1 of ESM. TMT- and taurine-affected HDO spectra (without the contributions of ND vibrations) decomposed into component OD bands with a physical significance are presented in Figs. 5 and 6. To aide interpretation of the component bands of HDO affected by the solutes, optimized structures of hydrated complexes of TMT and taurine (Fig. 7) were obtained from DFT calculations utilizing the polarizable continuum model (PCM). Intermolecular oxygen–oxygen distances (*R*_OO_ *)* from these structures were transformed to vibrational frequencies (*ν*_OD_) of OD bands using Eq. (1) (Berglund et al. 1978; Bratos et al. 2009):

1 $$\nu_{\text{OD}} = { 2727}\,{-}\,{ \exp }[ 1 6.0 1 \,{ }{-}\,{ 3}. 7 3(R_{\text{OO}} )],$$

with *R*_OO_ expressed in Å and *ν*_OD_ in cm^−1^.

In the case of TMT-affected HDO spectra, two component bands can be observed (Fig. 5). The calculated vibration frequencies of hydration structure of TMT (Fig. 7a) reveal that sulfonate oxygen atoms are non-equivalent in their interactions with the solvent with corresponding band positions at 2560 ± 2 and 2485 ± 4 cm^−1^. It is worth noting that a similar non-equivalency has been previously observed in the interactions of water with the carboxylate anions (Gojło et al. 2009) and the carboxylate oxygen atoms of amino acids (Panuszko et al. 2011, 2015). As in the case of the carboxylate group, unsymmetrical interaction of water molecules with the sulfonate group results from the orientation of the water molecules directly involved in this interaction. Two water molecules form a cooperative system, which plays a role as a non-equivalent proton donor with respect to both oxygen atoms of the sulfonate group (Gojło et al. 2009). In the affected spectra, increasing temperature correlates with a shift of both component bands towards higher wavenumbers, indicating weaker and longer hydrogen bonds between water molecules and the sulfonate oxygens. This shift is more pronounced for the band corresponding to stronger interactions: 2441 cm^−1^ at 25 °C to 2478 cm^−1^ at 75 °C. In addition, non-equivalent interaction of water molecules with the sulfonate group is maintained in the whole temperature range. The effect of methyl groups on the surrounding water molecules is not visible in the spectra of TMT-affected water, because the infrared water spectra corresponding to these groups resemble the bulk water (Panuszko et al. 2015).

Taurine-affected HDO spectra (Fig. 6) are characterized by the presence of four component bands. As in the case of spectra of water affected by TMT, two bands can be attributed to water–sulfonate group interactions. Analysis of calculated structures places them at 2557 and 2476 cm^−1^, while in the affected spectrum at 25 °C the bands are located at 2553 and 2465 cm^−1^, respectively. The intensity of the first band significantly decreases at higher temperatures (from 55 °C) and virtually disappears at 75 °C. This is due to the fact that the hydrogen bonds between water molecules interacting with both oxygen atoms of the sulfonate group of taurine disappear at higher temperatures, i.e., their non-equivalent interaction with the sulfonate group are destroyed. The disappearance of this band effectively separates the taurine-affected spectrum of water into two distinct hydration components. The band at ca. 2400 cm^−1^ corresponds to the interaction between two water molecules linking the sulfonate and amino groups. Such an interaction becomes more and more apparent with temperature increase. The small component band, at the high-wavenumbers position (2632 ± 10 cm^−1^), has been previously observed in spectra of water affected by urea (Panuszko et al. 2009), amino acids (Panuszko et al. 2011; 2015), and amides (Panuszko et al. 2008). Its presence is caused by the weak interaction of water molecules with one of hydrogen atom of amine group. It should be noted that non-equivalent interactions of water molecules with amino protons of taurine are also present in the calculated positions of the ND bands for hydrated structure (section S1.4, ESM). As can be seen from the above discussion, there is a good agreement between the calculated and the experimental band positions.

#### Structural and energetic characterization of the affected water

The solute-affected water spectrum gives valuable information about the energetic state of the hydrogen bonds of water and intermolecular distances between water molecules engaged in such interactions. Band shapes of TMT-affected HDO spectra (from Fig. 5) and taurine-affected spectra (from Fig. 6) were transformed into the oxygen–oxygen distance distribution function *P*(*R*_OO_) of the water molecules, according to Eq. (2) (Berglund et al. 1978; Bratos et al. 2009):

2 $$P\left( {{R_{{\rm{OO}}}}} \right) = {\rm{ }}[{\rm{16}}.0{\rm{1 }}{-}{\rm{ ln}}({\rm{2727 }}{-}{n_{{\rm{OD}}}})]/{\rm{3}}.{\rm{73}}$$

**Fig. 8:**
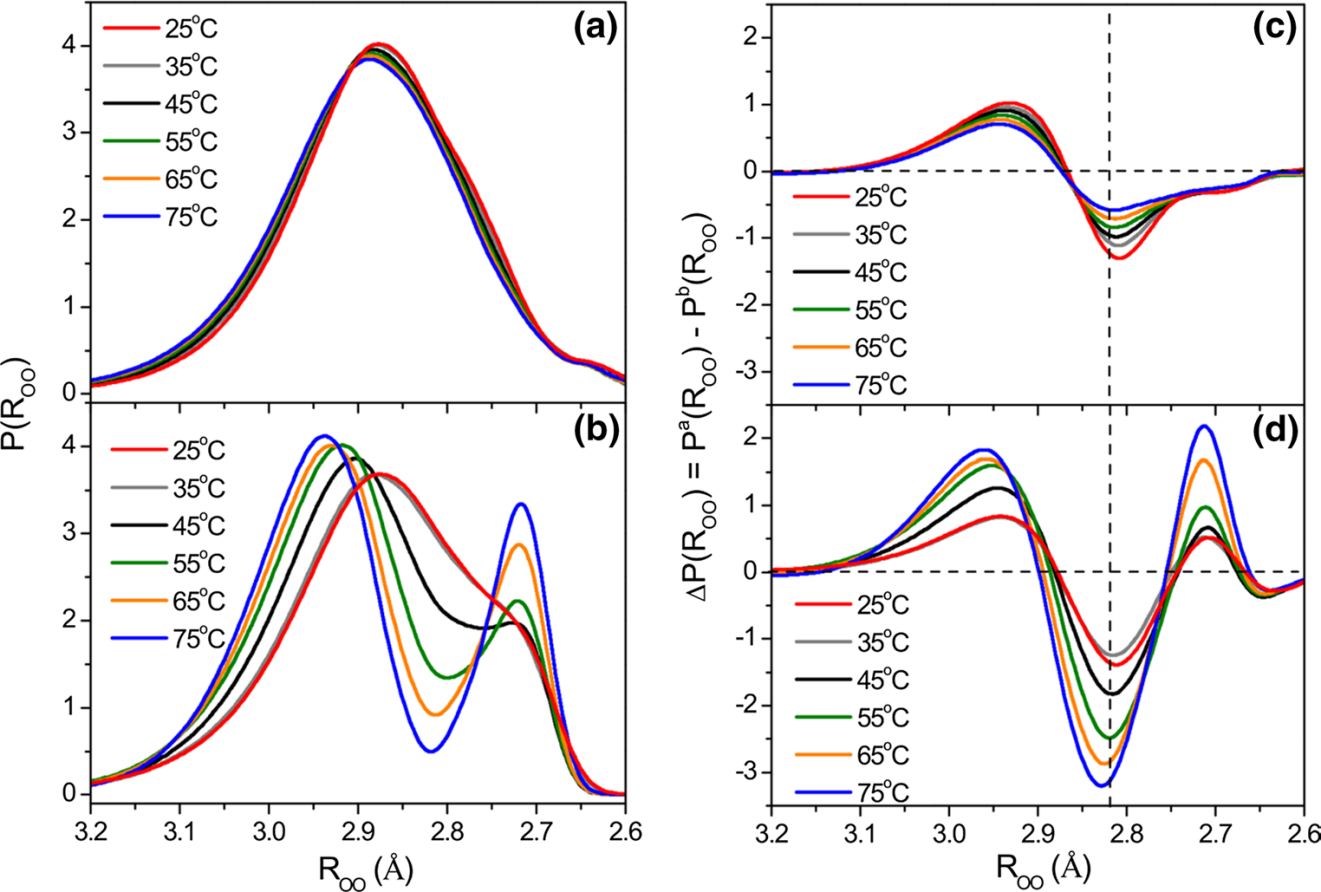
**a**, **b** Interatomic oxygen–oxygen distance distributions function derived from the HDO spectra affected by (a) TMT (Fig. 5) and **b** taurine (Fig. 6). **c**, **d** Differences between interatomic oxygen–oxygen distance distribution function of solute-affected water, *P* ^a^(*R*_OO_) and the “bulk” water, *P* ^b^(*R*_OO_) (Fig. S5, ESM) for **c** TMT and **d** taurine. The vertical dashed line corresponds to the value of the most probable oxygen–oxygen distance in bulk water at 25 °C (2.823 Å, see Table 3)

The obtained distance probability distributions are shown in Fig. 8a, b for TMT and taurine, respectively. Spectral parameters of affected HDO bands, together with the bulk HDO bands, for measured temperatures are summarized in Table 3, along with intermolecular oxygen–oxygen distances, *R*_OO_. The displacement of the most probable ($R_{\text{OO}}^{\text{o}}$) and mean ($R_{\text{OO}}^{\text{g}}$) distances towards higher values (i.e., longer oxygen–oxygen distances) with respect to the ones corresponding to pure water at a given temperature (Table 3) points out that water–water hydrogen bonds are weaker in the presence of these solutes. A comparison of the position of the center of gravity bands values, $\nu_{\text{OD}}^{\text{g}}$ (relates to the mean hydrogen bond energy of water molecules), for affected water and for bulk water (Table 3) suggests that water affected by these solutes forms on average weaker H-bonds than pure water in the whole temperature range.

**Table 3:** The parameters of HDO bands of bulk water (Fig. S2, ESM), water affected by taurine (Fig. 6), and water affected by TMT (Fig. 5), and the respective intermolecular oxygen–oxygen distances. *R*_*OO*_ errors have been estimated on the basis of the HDO bands position errors

*T* ^a^	*N* ^b^	$\nu_{\text{OD}}^{\text{o}}$ ^c^	$\nu_{\text{OD}}^{\text{g}}$ ^d^	*F*whh^e^	*I* ^f^	$R_{\text{OO}}^{\text{o}}$ ^g^	$R_{\text{OO}}^{\text{g}}$ ^h^
Bulk water spectrum							
25	–	2509 ± 2	2505 ± 2	162 ± 4	9874	2.823 ± 0.003	2.844 ± 0.003
35	–	2513 ± 2	2509 ± 2	165 ± 4	9487	2.836 ± 0.003	2.849 ± 0.003
45	–	2519 ± 2	2513 ± 2	167 ± 4	9120	2.836 ± 0.003	2.854 ± 0.003
55	–	2522 ± 2	2517 ± 2	166 ± 4	8789	2.838 ± 0.003	2.859 ± 0.003
65	–	2528 ± 2	2521 ± 2	170 ± 4	8457	2.844 ± 0.003	2.864 ± 0.003
75	–	2532 ± 2	2524 ± 2	169 ± 4	8159	2.849 ± 0.003	2.867 ± 0.003
Taurine—affected water spectrum							
25	11.3 ± 0.5	2548 ± 2	2524 ± 2	165 ± 4	7213	2.874 ± 0.003	2.869 ± 0.003
35	10.6 ± 0.5	2551 ± 2	2526 ± 2	163 ± 4	6387	2.882 ± 0.003	2.872 ± 0.003
45	8.6 ± 0.5	2563 ± 2	2542 ± 2	127 ± 4	4908	2.902 ± 0.003	2.892 ± 0.003
55	8.1 ± 0.5	2571 ± 2	2553 ± 2	110 ± 4	3613	2.918 ± 0.003	2.910 ± 0.003
65	7.4 ± 0.5	2576 ± 2	2557 ± 2	103 ± 4	2661	2.933 ± 0.003	2.915 ± 0.003
75	6.0 ± 0.5	2580 ± 2	2561 ± 2	94 ± 4	2553	2.933 ± 0.003	2.920 ± 0.003
TMT—affected water spectrum							
25	7.9 ± 0.5	2548 ± 2	2530 ± 2	153 ± 4	9690	2.877 ± 0.003	2.874 ± 0.003
35	7.5 ± 0.5	2549 ± 2	2532 ± 2	153 ± 4	9296	2.879 ± 0.003	2.879 ± 0.003
45	7.5 ± 0.5	2553 ± 2	2534 ± 2	152 ± 4	9160	2.882 ± 0.003	2.882 ± 0.003
55	7.9 ± 0.5	2553 ± 2	2536 ± 2	152 ± 4	8838	2.884 ± 0.003	2.884 ± 0.003
65	8.1 ± 0.5	2557 ± 2	2538 ± 2	156 ± 4	8616	2.887 ± 0.003	2.887 ± 0.003
75	8.1 ± 0.5	2557 ± 2	2540 ± 2	154 ± 4	8452	2.887 ± 0.003	2.889 ± 0.003

^a^Temperature (^°^C). ^b^Affected number, equal to the number of moles of water affected by one mole of solute. ^c^Band position at maximum (cm^−1^). ^d^Band position at gravity center (cm^−1^). ^e^Full width at half-height (cm^−1^). ^f^Integrated intensity (dm^3^ mol^−^1 cm^−1^). ^g^The most probable O^…^O distance (Å). ^h^Mean O^…^O distance (Å)

Considering the influence of temperature on the affected HDO spectra, it can be seen that temperature has only a slight effect on the water structure around TMT. Differences in the parameters of affected water spectra are small and the number of moles of water affected molecules (*N* values from Table 3) remains almost constant. Water molecules in the hydration sphere of taurine are more susceptible to temperature variations, which is reflected in changes of the band shapes of taurine-affected spectra as a function of temperature (Fig. 6). Differences in $\nu_{\text{OD}}^{\text{g}}$ or $R_{\text{OO}}^{\text{g}}$ values between affected water and bulk water (at a given temperature) increase with temperature and indicate that the taurine’s ability to weaken the water structure with the temperature increases.

It should be stressed that taurine, despite its protein stabilizing properties, weakens the water structure in their nearest surrounding in the whole temperature range, and affected-spectra parameters (like $\nu_{\text{OD}}^{\text{g}}$ or $R_{\text{OO}}^{\text{g}}$ from Table 3) categorize it as “structure breaking” solute. Therefore, “breaking” or “making” of water structure does not determine ability of osmolytes to “destabilizing” or “stabilizing” of protein structure (Ma et al. 2014; Zangi et al. 2009; Batchelor et al. 2004; Maclagan et al. 2004; Di Michele et al. 2006).

The interatomic oxygen–oxygen distance probability distributions (Fig. 8a) for TMT contain only one population of water molecules involved in hydrogen bonding with sulfonate oxygens. Analogous probability distributions *P*(*R*_OO_) for taurine (Fig. 8b) show that two types of water molecule populations can be distinguished in the hydration sphere of taurine: the first one refers to higher distance values (ca. 2.85–2.95 Å) and corresponds to the interaction of water molecules with sulfonate group, whereas the second one refers to the distance of ca. 2.72 Å and corresponds to the interactions of water molecules with amino group. This means that both functional groups of taurine have a different influence on surrounding water molecules: the sulfonate group weakens the structure of water while the amino group enhances the hydrogen bonds between water molecules. However, since the resultant water structure in the presence of taurine is weakened, we can state that the influence of the sulfonate group is predominant. It should be noted that such a division of water molecules into two populations in water affected by taurine becomes more pronounced with temperature. These two populations are not as clearly visible at lower temperatures (25 and 35 °C) as in higher ones because some of the water molecules are involved in hydrogen bonds with two functional groups of taurine simultaneously. In other words, this is due to overlap of the hydration spheres of the two functional groups. In effect, the hydration sphere of amino group has an enhancing effect on the hydration sphere of the sulfonate group. As a result, the weakening influence of the sulfonate group is diminished (the “breaking” properties of the sulfonate group decreases). This situation can be illustrated with the structure of hydrated complex of taurine (Fig. 7b). Water affected by taurine at lower temperatures is characterized by stronger hydrogen bonds than at higher temperatures. At higher temperatures hydrogen bonds between water molecules weaken. Both hydration spheres move away from each other and, in consequence, the enhancing influence of the hydration sphere of amino group on the hydration sphere of the sulfonate group is diminished.

The distance distribution function for “bulk” water, *P* ^b^(*R*_OO_) (Fig. S5, ESM), was subtracted from the distribution function of water affected by solute, *P* ^a^(*R*_OO_), to illustrate differences in intermolecular distances, Δ*P*(*R*_OO_), relative to bulk water at a given temperature. The operation was performed for all temperatures and the results are shown in Fig. 8c, d for TMT and taurine, respectively. The analysis of distance differences indicates that the population of very weak hydrogen bonds of water (*R*_OO_ > 2.9 Å) in the nearest surroundings of solutes increases, in comparison to bulk water. The population of water molecules with mean energy of hydrogen bonds (the population of water–water hydrogen bonds only slightly longer than and equal to the most probable distance in bulk water, Table 3) decreases, both in the presence of taurine and TMT. In addition, the hydration sphere of taurine is characterized by an increased population of stronger hydrogen bonds (distance values of ca. 2.72 Å), with respect to bulk water. This contribution increases with temperature.

## Conclusions

Despite structural similarity, taurine and TMT exert different effects on proteins. Calorimetric investigation indicates that TMT lowers protein thermal stability while taurine acts as a stabilizer. A new approach to the difference FTIR spectra method supported by chemometric analysis allowed to get a new insight into the net of interactions in such types of systems. ATR-FTIR studies and DFT calculations bring important information on differences in interactions of these compounds with proteins. The main one is the ability of taurine to interact directly with protein surface through ${\text{NH}}_{3}^{ + }$ group. This fact contradicts the hypothesis of preferential exclusion of stabilizing osmolytes. The sulfonate group of both taurine and TMT, though in principle able to create hydrogen bonds with surface proton donor groups, does not interact with proteins and influences them in indirect manner. Structural and energetic characteristics of water affected by these solutes indicate that in the presence of both the hydrogen bond network of water molecules is weakened. However, taurine produces in its surrounding two distinct populations of affected water: weakly bonded water molecules around the sulfonate group, and strongly bonded ones around amino group. Thus, we can conclude that ${\text{NH}}_{3}^{ + }$ group enhances water structure, while ${\text{SO}}_{3}^{ - }$ weakens it. It has to be stressed here that the water structure around protein is enhanced and such a water state is determined mainly by the protein backbone (Panuszko et al. 2012). The addition of taurine, which is oriented with its amino group towards the protein surface, the cooperativity of both water populations affected by taurine and protein enhances the hydrogen bond network around protein. In effect, its thermal stability also increases. The hydration sphere of TMT, with its weak net of hydrogen bonds, exerts structure-breaking effect on protein hydration water without direct interactions of TMT and protein. The energy of hydrogen bonds near the surface becomes lower in comparison to pure protein solution and the stability-determining protein hydration shell is being “dissolved”. In fact, a true osmophobic effect is observed for TMT. At lower concentrations, due to lack of any direct interactions with protein, it is preferentially excluded and protein stability actually increases. However, the detrimental properties of its water affected by its presence at some critical concentration (protein dependent) outweigh this positive effect.

The presented comprehensive studies are a step towards a better understanding of the role of solvent in the native conformation of proteins and provide valuable insight into the fundamental problem of protein stabilization in the presence of osmolytes. At the present stage, two general conclusions seem to be reasonable: (a) all the models approximating osmolytes in protein solutions as “hard spheres” are not sufficient (as in the case of taurine), and (b) the preferential exclusion theory is incomplete if water properties in such solutions are not considered (as in the case of TMT).

## Electronic supplementary material

Below is the link to the electronic supplementary material.

Supplementary material 1 (DOCX 1881 kb)
