# Supplementary material for: Taurine as a water structure breaker and protein stabilizer
Source: Amino Acids. 2017 Oct 17;50(1):125–40. doi: 10.1007/s00726-017-2499-x (PMC5762795; doi:10.1007/s00726-017-2499-x)
Supplement: Supplementary file 1 — Supplementary material 1 (DOCX 1881 kb) [file 726_2017_2499_MOESM1_ESM.docx]

**Electronic Supplementary Material**

**Taurine as a water structure breaker and protein stabilizer**

Piotr Bruździak,^* a^ Aneta Panuszko, ^a^ Emilia Kaczkowska, ^a^ Barnaba Piotrowski, ^a^ Anna Daghir,^a^ Sebastian Demkowicz,^b^ Janusz Stangret ^a^

^a^ *Department of Physical Chemistry, Chemical Faculty, Gdańsk University of Technology*

*Narutowicza 11/12, 80-233 Gdansk, Poland*

^b^ *Department of Organic Chemistry, Chemical Faculty, Gdańsk University of Technology*

*Narutowicza 11/12, 80-233 Gdansk, Poland*

* *Corresponding Author**E-mail:* [piotr.bruzdziak@pg.gda.pl](mailto:piotr.bruzdziak@pg.gda.pl)

**S1. The difference spectra method**

***S1.1. Sample preparation***

In the following process, each amount of added substance, be it solvent, solute or solution, was precisely weighted to ensure the composition of each solution was known. For both taurine and *N,N,N*-trimethyltaurine, a stock solution was prepared by dissolving the substance in deionized water. Portions of the stock solution were taken and diluted with deionized water to obtain a series of solutions with desired concentrations. Next, for each concentration, the solutions were divided into two parts. D_2_O (isotopic purity 99.9%, *Aldrich*) was added to one of these parts in the amount of 4% (by weight) of water in the solution. This, in accordance with the equilibrium constant of the reaction (H_2_O + D_2_O = 2HDO, *K* ≈ 4), should result in a quantitative transformation of D_2_O into HDO. To obtain a reference solution, a molarly equivalent amount of H_2_O was added to the another part of the solution to equalize the concentrations.

Solution densities were measured using an Anton Paar DMA 5000 densitometer at all studied temperatures (25, 35, 45, 55, 65 and 75 °C within a margin of 0.001 °C).

***S1.2. The difference spectra method***

The difference spectra method is based on an assumption that one can divide the water in a solution into two additive populations: “bulk” water, identical to pure water, and “affected” water, the qualities of which have been affected through interactions with the solute. Since both populations contribute to the spectrum of water in a solution it is possible to isolate the spectrum of affected water in a hypothetical infinite dilution by utilizing the following Equation (1):

 (1)

where *ε_a_* and *ε_b_* are, accordingly, the molar absorption coefficients of affected and bulk water, *N* is the number of moles of water affected by one mole of solute, *M* is the molar mass of water (with the isotopic composition taken into account, as in the case of our solutions with added D_2_O) (kg⋅mol^-1^) and *m* is the molality of the solute (mol⋅kg^-1^). *(∂ε/∂m)_m=0_* is the derivative of the solvent’s molar absorption coefficient with respect to molality at the limit of infinite dilution.

Calculation of the aforementioned derivative is the first step in our approach. A series of normalized spectra of concentration-varying solutions is fitted with a regression curve (*ε* as a function of molality), either linear or quadratic, at each wavenumber. The derivative of this fit at *m* = 0 is used.

The *N* parameter, also called the “affected number”, cannot be obtained independently of *ε_a_*. To obtain both, we utilize a method based on curve fitting. “Test” affected spectra are obtained for different values of *N*. These are then fitted with a baseline, several analytical bands with a starting shape of the Gaussian and Lorentzian product peak, and the bulk water spectrum. The number of analytical bands is chosen to be the lowest needed to ensure an adequate fit. All their parameters, including position, shape, band half-width and intensity, are set to vary. The bulk water spectrum peak has all its parameters except intensity set to fixed. The “true” value of *N* is determined to be the highest one for which the bulk water peak band has a negligible contribution to the fit (usually below 0.5% of the total integral intensity of the test spectrum). As a result, the value of *N* with ca. 5% error and the affected water spectrum are simultaneously determined.

The detailed procedure of spectral data analysis toward extraction of the solute-affected water spectrum was described previously.^1-4^ This method was used extensively in our laboratory *inter alia* to study the hydration of aqueous solutions of molecules of biological importance such as osmolytes and proteins.^5-7^ The effectiveness of this method has been confirmed by molecular dynamics simulations^5,7,8^ as well as by chemometric analysis of HDO spectra in aqueous solutions.^4^

***S1.3. Determination of taurine- and N,N,N-trimethyltaurine-affected water spectra.***

For each temperature and solute, a detailed analysis of solution spectra was performed. A normalized HDO spectrum was obtained from each spectrum of a HDO-containing solution by subtracting its reference solution (containing no HDO). These spectra served to calculate an approximation of the molar absorption coefficient vs. molality at every wavenumber, from which the derivative spectrum *(∂ε/∂m)_m=0_* was obtained (separately for each temperature and solute combination). The approximation was achieved through the least-squares linear regression method. Figure S1 presents all derivative spectra obtained as detailed above.

For each series of spectra, the derivative and the bulk HDO spectrum (Fig. S2) (solute molality *m*=0) were utilized to determine the affected HDO spectrum and affected number (*N*) through the curve fitting method as described in the previous section. These spectra are shown in Figure S3.

***S1.4. Isolation of the ND band***

The taurine-affected spectra contain a contribution of the vibrational band of ν_ND_. This is due to the presence of amino hydrogens that can be exchanged for deuterium due to interaction with the deuterated solvent. For the affected spectrum to be a reliable source of information on solvent interactions, this additional band must be subtracted. Figure S4 illustrates the process of isolation and subtraction of the ND band step by step for the taurine solution series at 25 °C. TMT-affected spectra do not contain the ND band due to the amino group being fully methylated. To find the wavenumber range within which the ND band is contained, the TMT-affected spectrum, scaled to have the same peak intensity (Fig. S4a), was subtracted from the taurine-affected spectrum (Fig. S4b). The ND stretching vibration region, which is additional relative to the TMT-affected HDO, was fitted with analytical bands (Fig. S4c). Identification of ND band positions was made on the basis of the calculated ν_ND_ (2260, 2267 and 2341 cm^-1^) and ν_OD_ values of vibrational frequencies for optimized structure of hydrated complex of taurine shown in Figure 7b in the main part of the manuscript. The component bands ascribed to ND vibrations were combined into one band (Fig. S4d). Taurine-affected spectrum was again fitted with the shape and starting position of the previously isolated ND band. All parameters of this band were allowed to change during fitting procedure except the shape. The adequate approximation of the spectrum required additional analytical band components (Gaussian and Lorentzian product function shape, all parameters were variable during fitting). Figure S4e presents the decomposition of the taurine-affected water spectrum at 25 °C into component OD and ND bands. Figure S4f shows taurine-affected spectra for all studied temperatures after subtracting the ND contribution.

***S1.5. Interpretation of solute–affected water spectra.***

The position of the OD water band has a direct relationship with the energy of the hydrogen bonds. On the basis of the Badger−Bauer’s rule,^9^ which states that the energy of hydrogen bonds of water molecules changes proportionally to the shift of OD band position, it is possible to define the energetic state of water molecules in hydration spheres of solutes. The position of the HDO absorption band maximum (*ν^o^_OD_*) refers to the most probable hydrogen bond energy, and the position of the band gravity center (*ν^g^_OD_*) relates to the mean energy of water hydrogen bonds.

In the presence of water “structure - making” solutes the value of the centre of gravity of OD band is shifted towards lower wavenumbers with respect to the corresponding position for pure water. It is accompanied by the shortening of oxygen - oxygen intermolecular distance. For the “structure - breaking” solutes it is shifted towards higher wavenumbers with increasing of the oxygen - oxygen intermolecular distance.

**S2. Isolation of ATR-FTIR spectra of osmolyte.**

A variant of the difference spectra method was employed to isolate spectra of taurine affected by the presence of proteins. In our approach we assumed that taurine or TMT in protein solutions can be divided into two separate spectral individuals: bulk molecules interacting only with water molecules and affected molecules interacting directly or indirectly with proteins. The first spectral individual is known (i.e. it is the spectrum of taurine or TMT in water), while the second one is to be determined. The basic equation of the method allows to calculate the ATR molar absorbance at any given wavenumber (i.e., also a complete spectrum if these ATR absorbances are calculated in a range of wavenumbers) when the number of affected taurine molecules, *N*, is known:

$A_{ATR,aff}=\frac{1}{NM}\cdot\left( \frac{dA_{ATR}}{dm} \right)_{m\to0}+A_{ATR,bulk}$ (2)

where *_ATR,aff_* is an affected ATR absorbance (per 1 mol of the solute) at a given wavenumber, *_ATR,bulk_* is a bulk ATR absorbance (per 1 mol of the solute) at the given wavenumber, *M* is the molar weight of an affected individual – taurine or TMT, *(d_ATR_/dm)_m→0_* is the derivative in the infinite dilution of protein limit of the molar ATR absorbance at a given wavenumber vs. protein molality (mol∙kg^-1^ of taurine). The equation is a variation of the formula used to determine “affected” spectra of HDO in solutions of various compounds (as in section S1). However, here water serves only as the environment of observed interactions.

The equation contains two unknowns: *_ATR,aff_* (or a “spectrum” if *_ATR,aff_* is calculated for a range of wavenumbers) and *N*. The only way to solve it is to assume *N* and calculate *_ATR,aff_*. The resultant “affected” spectrum is tested if it can be accepted as “true affected” spectrum. Various methods exist to fulfill this task. Here, we used a chemometric approach, described in details in refs. 3 and 4. The affected spectrum of taurine or TMT contains spectral features only of molecules interacting (directly or indirectly) with proteins and gives information on the character and strength of such interactions.

**S3. Calculation of spectroscopy-derived preferential interaction coefficients.**

The calculation of the number of affected molecules, *N*, gives a general view on interactions in analyzed system. However, the number has to be compared with the bulk solution of the compound to understand if such interactions are favorable, unfavorable or neutral in comparison to the general number of compound molecules in protein solutions. Equation (3) gives an estimation of the preferential interactions or exclusion of such molecules, here by the example of taurine, from the protein surrounding:

$P^{S}=\frac{N_{tau}}{N_{H_{2}O}-N_{tau}}\cdot\left( \frac{n_{tau}}{n_{H_{2}O}} \right)^{-1}$ (3)

Here *N_tau_* is the number of taurine molecules affected by one protein molecule, *N_H2O_* is the number of water molecules affected by the same protein (these water molecules are most likely to interact directly with the protein surface and are exchanged to osmolyte molecules in ternary solutions). *n_tau_* and *n_H2O_* are the overall numbers of moles of taurine and water in a given solution, respectively. *P^s^* indicates if more taurine or water is accumulated in the vicinity of proteins in comparison to initial concentrations of these components in a system. If the factor reaches values higher than 1 the osmolyte is not excluded from the surface. In such a case, there is a direct interaction between analyzed molecule and protein. In our calculations we assume that one water molecule, directly interacting with lysozyme via hydrogen bonding, is replaced by one molecule of taurine. When the value of *P^s^* is lower than 1 this indicates an exclusion of osmolyte from the protein surface. No appropriate hydration data for ubiquitin was available, thus we had to predict it from the protein solvent accessible surface area (*SASA^Ubi^* = 4641 Å^2^), with lysozyme surface area (*SASA^Lys^* = 6496 Å^2^) and lysozyme hydration number *N_H2O_* = 385 as references.^7^ Solvent accessible areas were estimated using PyMOL software with 1AKI and 4Y1H pdb structures (for lysozyme and ubiquitin, respectively) and water molecule radius of 1.4 Å. In the case of ubiquitin, the *N_H2O_* value (277) was estimated on the basis of the following proportion: *N*^ubi^_H2O_/*SASA*^ubi^ = *N*^lys^_H2O_/*SASA*^lys^.

**S4. Chemometric decomposition of affected spectra**

The chemometric method, described in refs. 3 and 4and employed to determine the shape of protein-affected taurine spectra in the paper, indicates not one but multiple possible pairs of “affected” numbers *N* and corresponding ”affected” spectra. The pair of the lowest *N* number (*N_1_*) and a physically meaningful “affected” spectrum should be considered as those describing the most affected state of analyzed molecule. Higher *N* numbers (*N_2_, N_3_... N_i_*) correspond to those molecules which are also affected to a lesser extent: for instance, the *N_2_* number corresponds to the sum of *N_1_* molecules which are affected to the highest degree and the number of molecules affected to a lesser extent, i.e. (*N_2_-N_1_*). In our work, we limit our analysis to first two pairs of *N* and spectrum because they cover most important information on interactions in the system.

The method, however, is not perfect, because of mathematical characteristics of spectral data and its inevitable uncertainty, and results of analysis can be scattered within several spectral series. Here, we propose a scheme of steps facilitating the determination or unification of “true” affected numbers and “affected” spectra if several spectral series of analyzed systems are available.

In our case, a single system comprised of aqueous solutions of taurine with a variable concentration of protein (i.e. affecting agent). Here only taurine-ubiquitin system will be described. Four of such series were collected with taurine concentration (yet still fixed in a single series). Each single series was subject to chemometric analysis (as described in our previous papers) and for each of them affected numbers and spectra were obtained (see results in Figure S6 for clarity).

We chose first two *N* numbers for each of four series and calculated an appropriate set of affected spectra of taurine. The lower *N* numbers, *N_1_* – corresponding to the highly affected *N* numbers, raised our objections and had to be determined with a method described below. Especially, the last series corresponding to 0.4 mol/kg of taurine exhibited almost a two-fold increase of *N* value compared to the 0.3 mol/kg series, and calculated spectroscopic preferential coefficients did not form a reasonable dependence. The higher one *N_2_*, in the case of ubiquitin, fell into place and was our first approximation of *N* value. With the set of affected taurine spectra corresponding the higher *N* numbers (*N_2_* value of each series) and to different taurine concentrations, we performed the following series of steps which allowed us to predict proper values of highly affected *N* numbers (the lower ones) and gave us a wider picture of protein-taurine relations in aqueous solutions:

1. The set of affected taurine spectra was first the subject of Principal Factor Analysis (PFA) giving the number of principal spectral components; it should be compatible with a selected sequence of *N* numbers (i.e. here we predicted that it would comprise of two principal factor corresponding to highly and less affected spectra, respectively, because we chose to analyze the second *N_2_* value in each series);
2. Next, Spectral Factor Isolation algorithm was applied to determine relative concentration profiles of principal factors composing the analyzed set of spectra (C*_N1_*, C*_N2_*... C*_Ni_*; the algorithm gives also the spectral shapes of the factors, which sometimes can be helpful yet cannot be taken as real spectra);
3. Each spectrum in the input set corresponded to a particular *N* affected number; the number could be decomposed into several contributions according to the relative concentrations from point 2 using a simple proportion: C_N1_:C_N2_:... = *N_1_:N_2_*:... with C_total_ being the sum of relative concentrations from point 2 for a single series and *N_total_* being the affected number corresponding to the series.
4. Having the *N_1_, N_2_*, ... numbers for each series it is easy to determine the shape of the highly affected spectrum of taurine* *– it can be directly calculated with Equation (2).
5. If the number of principal factors equals to 2, as in the case of our systems, the less affected spectrum should be simply the difference between the initial affected spectrum, corresponding to *N_total_*, and the highly affected one from point 4:

*_less aff_= _total aff_ − a∙_highly aff_*. However, the information on intensities of all these spectra is lost at various steps of analysis, and the subtraction factor *a* is unknown. Again, such an equation with two unknowns can be solved with the difference spectra method. The equation is very similar to Equation (2) and a similar procedure can be applied, i.e. a series of test less affected spectra (corresponding to different subtraction factors) is a subject of chemometric method of affected spectra determination.

**FIGURES**

**Fig. S1.** Derivatives at infinite dilution limit *(∂ε/∂m)_m=0_* for solute-affected HDO spectra in the OD stretching region for solutions of (a) TMT and (b) taurine for all studied temperatures.

**Fig. S2.** The bulk HDO spectra as a function of temperature.

**Fig. S3.** Solute-affected HDO spectra in the OD stretching region for (a) TMT and (b) taurine for all studied temperatures. Taurine-affected spectra shown before ND-band isolation and subtraction.

**Fig. S4.** Isolation and subtraction of the ND band from taurine-affected spectrum at 25 °C. (a) Solute-affected spectra of taurine and TMT scaled to the same maximum absorption value. (b) Subtraction of TMT-affected spectrum from taurine-affected spectrum (subtraction factor = 1). Region containing ND band contribution presented with green outline. (c) Decomposition of region containing ND band contribution (black) into component bands of OD and ND stretching. (d) Sum of ND stretching component bands from Fig. S4c. (e) Decomposition of taurine-affected spectrum (black) into component OD bands and isolated ND band. (f) Taurine-affected spectra after subtraction of ND vibration contribution for all temperatures.

**Fig. S5.** Interatomic oxygen-oxygen distance distributions function derived from the bulk HDO spectra.

*N_1_*

*N_1_*

*N_1_*

*N_2_*

*N_2_*

*N_2_*

*N_1_*

*N_2_*

*N*, affected number

Window width

0.1 0.2 0.3 0.4

mol_Tau_/kg_H2O_ mol_Tau_/kg _H2O_ mol_Tau_/kg _H2O_ mol_Tau_/kg _H2O_

**Fig. S6.** Results of chemometric analysis for four single sets of spectra of taurine in the presence of ubiquitin. Each panel corresponds to a fixed taurine molality. Arrows indicate first two *N* numbers. Each *N* number has a corresponding “affected spectrum”. In this set of data there are four spectral series with two *N* values.

77.4±0.1 ^o^C


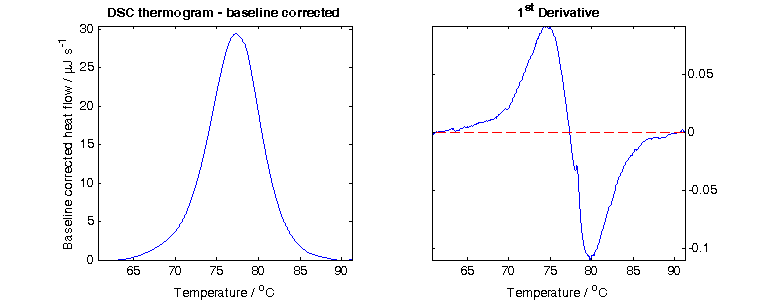


**Fig. S7.** Baseline corrected DSC thermogram of **lysozyme** and its first derivative indicating melting temperature.

77.8±0.1 ^o^C


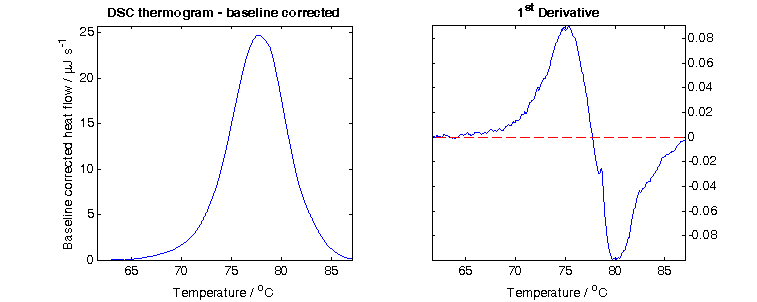


**Fig. S8.** Baseline corrected DSC thermogram of **lysozyme in the 0.1 mol·kg^-1^**solution of taurineand its first derivative indicating melting temperature.

78.1±0.1 ^o^C


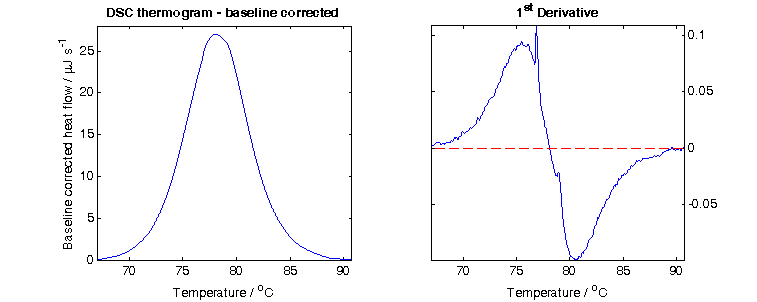


**Fig. S9.** Baseline corrected DSC thermogram of **lysozyme in the 0.2 mol·kg^-1^**solution of taurineand its first derivative indicating melting temperature.

78.3±0.1 ^o^C


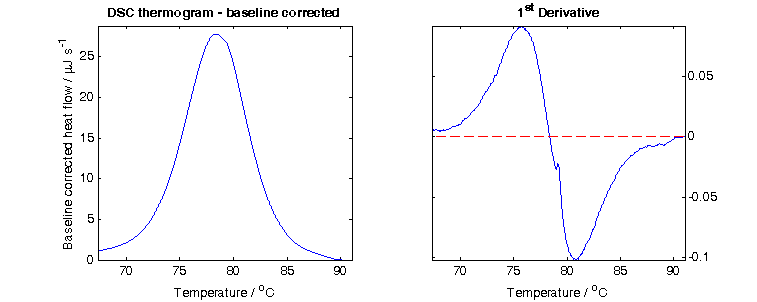


**Fig. S10.** Baseline corrected DSC thermogram of **lysozyme in the 0.3 mol·kg^-1^**solution of taurineand its first derivative indicating melting temperature.

78.7±0.1 ^o^C


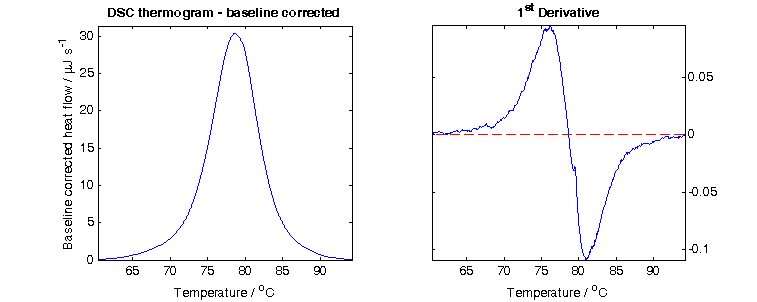


**Fig. S11.** Baseline corrected DSC thermogram of **lysozyme in the 0.4 mol·kg^-1^**solution of taurineand its first derivative indicating melting temperature.

77.8±0.1 ^o^C


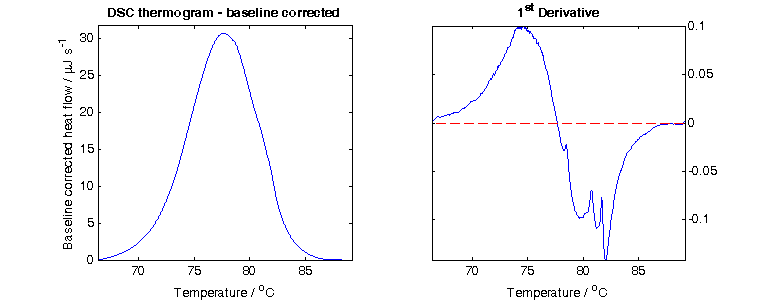


**Fig. S12.** Baseline corrected DSC thermogram of **lysozyme in the 0.025 mol·kg^-1^**solution of TMTand its first derivative indicating melting temperature.

77.2±0.1 ^o^C


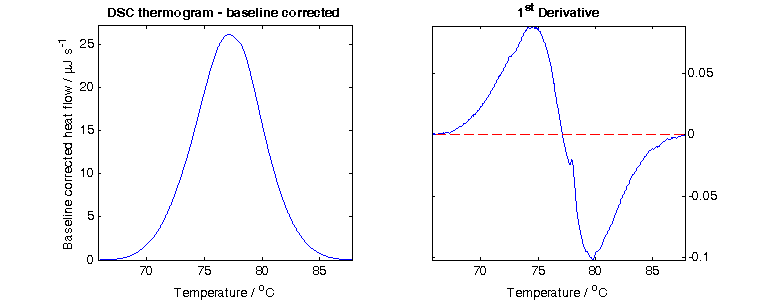


**Fig. S13.** Baseline corrected DSC thermogram of **lysozyme in the 0.1 mol·kg^-1^**solution of TMTand its first derivative indicating melting temperature.

76.0±0.1 ^o^C


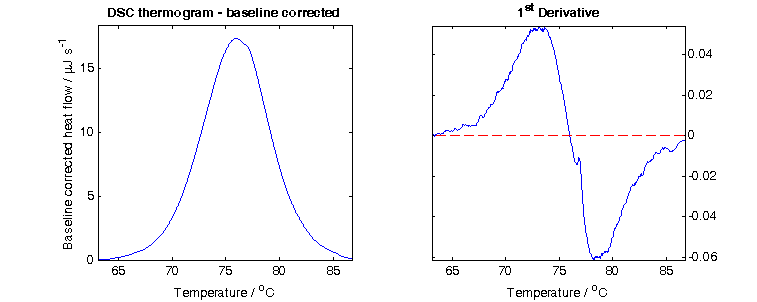


**Fig. S14.** Baseline corrected DSC thermogram of **lysozyme in the 0.2 mol·kg^-1^**solution of TMTand its first derivative indicating melting temperature.

74.9±0.1 ^o^C


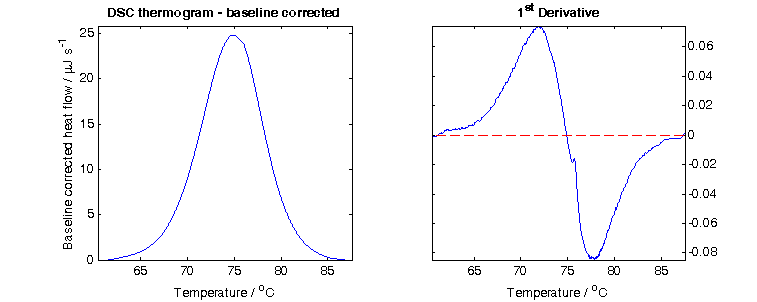


**Fig. S15.** Baseline corrected DSC thermogram of **lysozyme in the 0.3 mol·kg^-1^**solution of TMTand its first derivative indicating melting temperature.

73.9±0.1 ^o^C


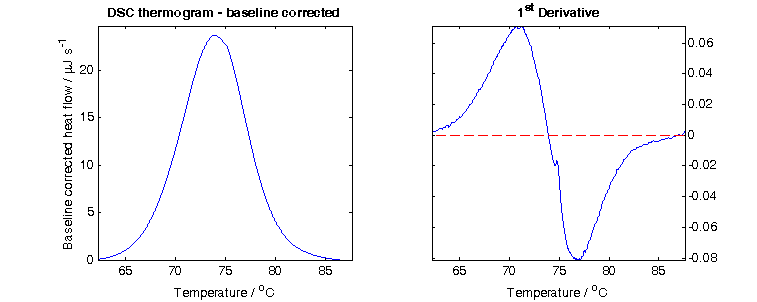


**Fig. S16.** Baseline corrected DSC thermogram of **lysozyme in the 0.4 mol·kg^-1^**solution of TMTand its first derivative indicating melting temperature.

95.7±0.5 ^o^C


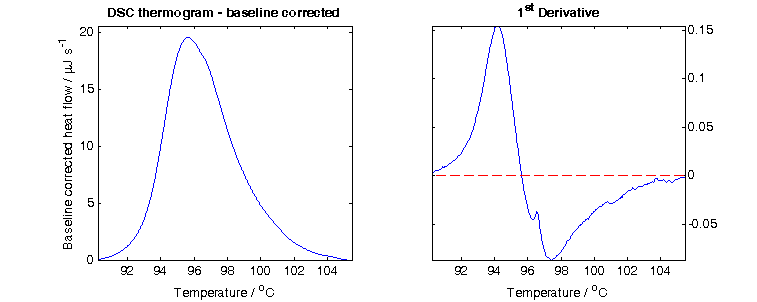


**Fig. S17.** Baseline corrected DSC thermogram of **ubiquitin** and its first derivative indicating melting temperature.

98.4±0.5 ^o^C


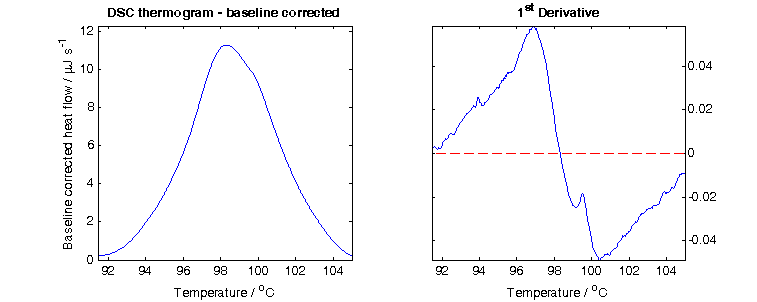


**Fig. S18.** Baseline corrected DSC thermogram of **ubiquitin in the 0.05 mol·kg^-1^**solution of taurineand its first derivative indicating melting temperature.

99.5±0.5 ^o^C


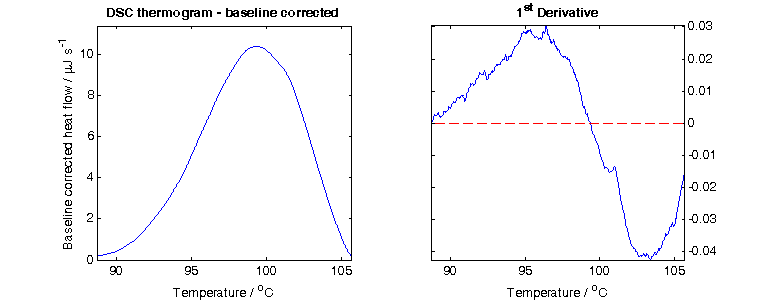


**Fig. S19.** Baseline corrected DSC thermogram of **ubiquitin in the 0.1 mol·kg^-1^**solution of taurineand its first derivative indicating melting temperature.

98.7±0.5 ^o^C


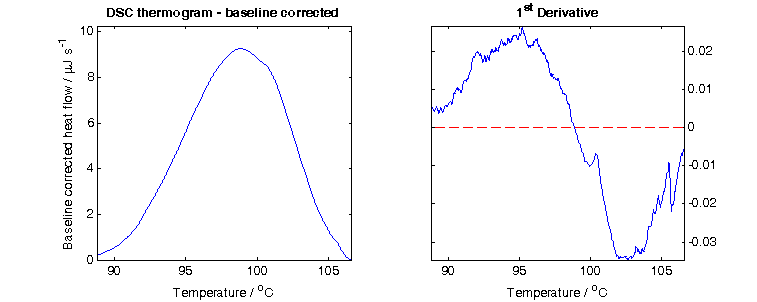


**Fig. S20.** Baseline corrected DSC thermogram of **ubiquitin in the 0.2 mol·kg^-1^**solution of taurineand its first derivative indicating melting temperature.

98.7±0.5 ^o^C


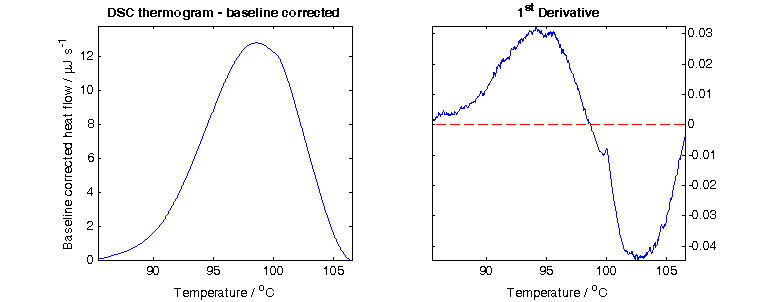


**Fig. S21.** Baseline corrected DSC thermogram of **ubiquitin in the 0.3 mol·kg^-1^**solution of taurineand its first derivative indicating melting temperature.

98.2±0.5 ^o^C


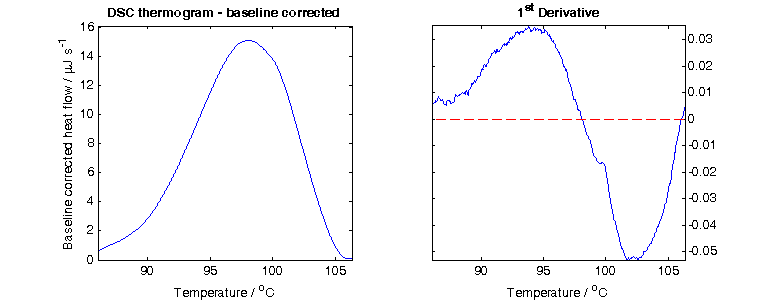


**Fig. S22.** Baseline corrected DSC thermogram of **ubiquitin in the 0.4 mol·kg^-1^** solution of taurineand its first derivative indicating melting temperature.

97.2±0.5 ^o^C


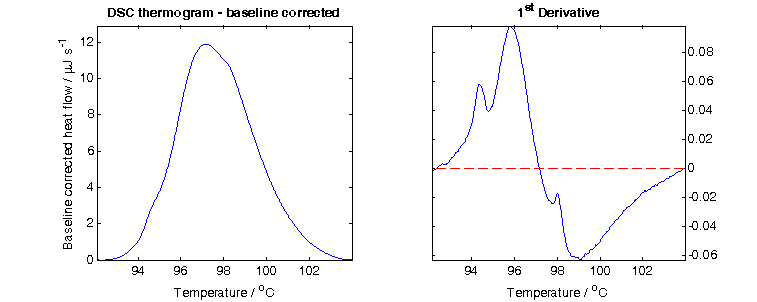


**Fig. S23.** Baseline corrected DSC thermogram of **ubiquitin in the 0.05 mol·kg^-1^**solution of TMTand its first derivative indicating melting temperature.

98.6±0.5 ^o^C


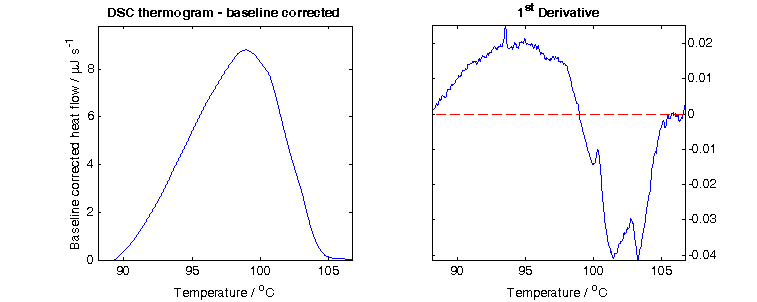


**Fig. S24.** Baseline corrected DSC thermogram of **ubiquitin in the 0.1 mol·kg^-1^**solution of TMTand its first derivative indicating melting temperature.

95.7±0.5 ^o^C


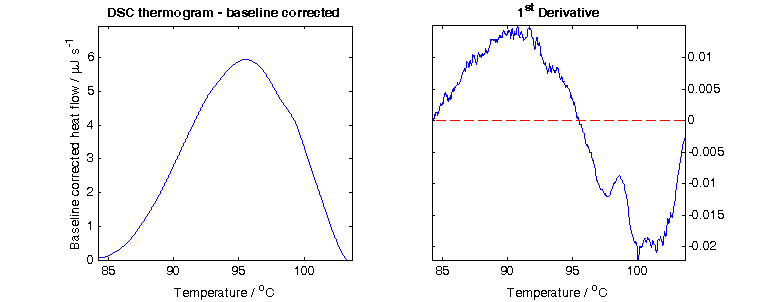


**Fig. S25.** Baseline corrected DSC thermogram of **ubiquitin in the 0.2 mol·kg^-1^**solution of TMTand its first derivative indicating melting temperature.

92.1±0.5 ^o^C


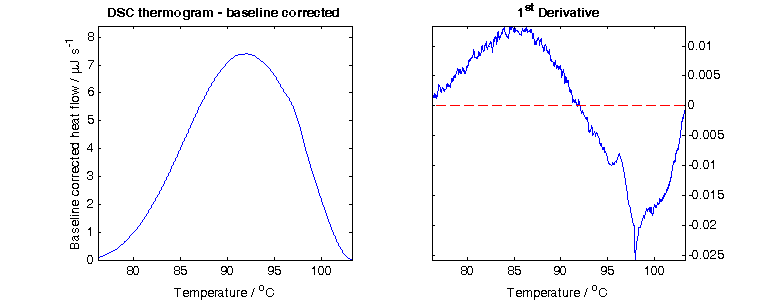


**Fig. S26.** Baseline corrected DSC thermogram of **ubiquitin in the 0.3 mol·kg^-1^**solution of TMTand its first derivative indicating melting temperature.

88.4±0.5 ^o^C


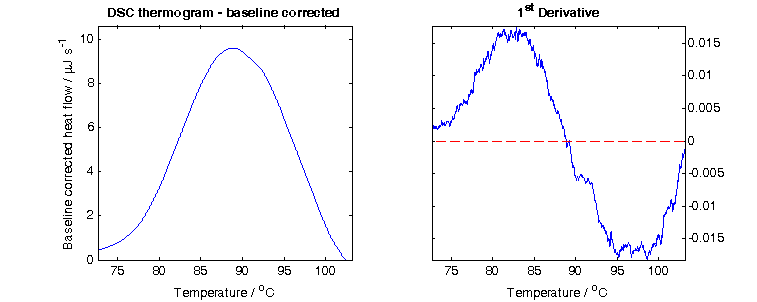


**Fig. S27.** Baseline corrected DSC thermogram of **ubiquitin in the 0.4 mol·kg^-1^**solution of TMTand its first derivative indicating melting temperature.

**REFERENCES**

(1) Stangret, J. Solute-Affected Vibrational-Spectra of Water in Ca(ClO_4_)_2_ Aqueous Solutions. *Spectrosc. Lett.* **1988**, *21*, 369-381.

(2) Śmiechowski, M.; Stangret, J. Vibrational Spectroscopy of Semiheavy Water (HDO) as a Probe of Solute Hydration. *Pure Appl. Chem.* **2010**, *82*, 1869-1887.

(3) Bruździak, P.; Rakowska, P. W.; Stangret, J. Chemometric Method of Spectra Analysis Leading to Isolation of Lysozyme and CtDNA Spectra Affected by Osmolytes. *Appl. Spectrosc.* **2012**, *66*, 1302-1310.

(4) Bruździak, P.; Panuszko, A.; Stangret, J. Chemometric Determination of Solute-Affected Solvent Vibrational Spectra as a Superior Way of Information Extraction on Solute Solvation Phenomena. *Vib. Spectrosc.* **2010**, *54*, 65-71.

(5) Panuszko, A.; Bruździak, P.; Zielkiewicz, J.; Wyrzykowski, D.; Stangret, J. Effects of Urea and Trimethylamine-N-oxide on the Properties of Water and the Secondary Structure of Hen Egg White Lysozyme. *J. Phys. Chem. B* **2009**, *113*, 14797-14809.

(6) Panuszko, A.; Śmiechowski, M.; Stangret, J. Fourier Transform Infrared Spectroscopic and Theoretical Study of Water Interactions with Glycine and its N-Methylated Derivatives. *J. Chem. Phys.* **2011**, *134*, 115104(1)-115104(9).

(7) Panuszko, A.; Wojciechowski, M.; Bruździak, P.; Rakowska, P. W.; Stangret, J. Characteristics of Hydration Water around Hen Egg Lysozyme as the Protein Model in Aqueous Solution. FTIR Spectroscopy and Molecular Dynamics Simulation. *Phys. Chem. Chem. Phys.* **2012**, *14*, 15765-15773.

(8) Panuszko, A.; Gojlo, E.; Zielkiewicz, J.; Smiechowski, M.; Krakowiak, J.; Stangret, J. Hydration of Simple Amides. FTIR Spectra of HDO and Theoretical Studies.  *J. Phys. Chem. B* **2008**, *112*, 2483-2493.

(9) Badger, R. M.; Bauer, S. H. Spectroscopic Studies of the Hydrogen Bond. II. The Shift of the O–H Vibrational Frequency in the Formation of the Hydrogen Bond. *J. Chem. Phys.* **1937**, *5*, 839-851.
